# The interaction of diet, alcohol, genetic predisposition, and the risk of breast cancer: a cohort study from the UK Biobank

**DOI:** 10.1007/s00394-023-03269-8

**Published:** 2023-11-01

**Authors:** Pingxiu Zhu, Yanyu Zhang, Qianni Chen, Wenji Qiu, Minhui Chen, Lihua Xue, Moufeng Lin, Haomin Yang

**Affiliations:** 1 Department of Epidemiology and Health Statistics, School of Public Health, Fujian Medical University, Xuefu North Road 1, University Town, Fuzhou, 350122 China; 2 Department of Ultrasonography, Fuqing City Hospital Affiliated to Fujian Medical University, Fuqing, China; 3 School of Health Management, Fujian Medical University, Fuzhou, 350122 China; 4 No. 5 Hospital of Fuqing City, Fuzhou, 350319 China; 5 Department of Medical Epidemiology and Biostatistics, Karolinska Institutet, Nobels Väg 12A, 171 77 Stockholm, Sweden

**Keywords:** Dietary factors, Alcohol, Polygenic risk score, Breast cancer

## Abstract

**Background:**

Dietary factors have consistently been associated with breast cancer risk. However, there is limited evidence regarding their associations in women with different genetic susceptibility to breast cancer, and their interaction with alcohol consumption is also not well understood.

**Methods:**

We analyzed data from 261,853 female participants in the UK Biobank. Multivariable adjusted Cox proportional hazards models were used to estimate hazard ratios (HR) and 95% confidence intervals (CI) for associations between dietary factors and breast cancer risk. Additionally, we assessed the interaction of dietary factors with alcohol consumption and polygenic risk score (PRS) for breast cancer.

**Results:**

A moderately higher risk of breast cancer was associated with the consumption of processed meat (HR = 1.10, 95% CI 1.03, 1.18, *p*-trend = 0.016). Higher intake of raw vegetables and fresh fruits, and adherence to a healthy dietary pattern were inversely associated with breast cancer risk [HR (95% CI):0.93 (0.88–0.99), 0.87 (0.81, 0.93) and 0.93 (0.86–1.00), *p* for trend: 0.025, < 0.001, and 0.041, respectively]. Furthermore, a borderline significant interaction was found between alcohol consumption and the intake of processed meat with regard to breast cancer risk (*P* for interaction = 0.065). No multiplicative interaction was observed between dietary factors and PRS.

**Conclusion:**

Processed meat was positively associated with breast cancer risk, and vegetables, fruits, and healthy dietary patterns were negatively associated with breast cancer risk. We found no strong interaction of dietary factors with alcohol consumption and genetic predisposition for risk of breast cancer.

**Supplementary Information:**

The online version contains supplementary material available at 10.1007/s00394-023-03269-8.

## Introduction

Breast cancer is the most prevalent cancer in women worldwide and the second most common cause of cancer mortality [1, 2]. With the growing incidence of breast cancer around the world [3], it is crucial to identify lifestyle risk factors that may help reduce the risk of breast cancer, including physical activity, breastfeeding, and consumption of healthy food.

Population-based studies have reported several dietary patterns that are associated with the risk of breast cancer [4], including Healthy/Mediterranean patterns or based on dietary guidelines (e.g., the Healthy Eating Score) [5], while specific foods such as oily fish, fruits, and vegetables may help reduce the risk of developing breast cancer [6]. However, previous evidence regarding the associations between different types of meat consumption and breast cancer risk has been inconclusive [7, 8], which could be attributed to differences in study design or the use of specific subgroups of women. Moreover, despite the known association between alcohol intake and breast cancer [9, 10], no study to date has assessed their synergistic effect with food on the risk of breast cancer, which could result in targeted interventions for women and increase the cost-effectiveness of interventions.

Besides the interaction with alcohol intake, the association between dietary factors and breast cancer could also be influenced by genetic factors. Recent GWAS studies have revealed 313 SNPs associated with breast cancer risk that could be used as a tool for risk stratification [11]. However, it is still unclear whether the associations with dietary factors differ according to different genetic predispositions to breast cancer, which is important for personalized prevention.

The aim of this study was to evaluate the association between dietary factors and the risk of breast cancer and to investigate whether the association was influenced by alcohol consumption, particularly when taken with meals. We also examined the association between dietary factors and breast cancer risk taking into account genetic susceptibility measured by polygenic risk score.

## Methods

### Study population, exposure, and outcome

Between 2006 and 2010, a total of 503,317 participants agreed to participate in the baseline assessment of the UK Biobank study, of which 273,382 were women [12]. Participants were followed up from the date of enrollment until the date of diagnosis of breast cancer, withdrawal from the study, death, loss of follow-up, or the end of follow-up (December 31, 2019), whichever occurred first. Information on breast cancer diagnosis was obtained by linking the cohort to the National Health Service (NHS) Digital for England and Wales and NHS Scotland, using unique personal identification numbers. The ICD-10 code C50 and ICD-9 code 174 were used to identify breast cancer diagnoses in the cancer register. Women with breast cancer before participating in the UK Biobank were excluded from the analysis. The date of death was retrieved from death certificates held by the NHS Information Center and the NHS Central Register. The study was approved by The National Information Governance Board for Health and Social Care and the NHS North West Multicentre Research Ethics Committee (06/MRE08/65). The participants gave informed consent at the baseline and agreed to be followed up via data linkage.

### Diet group classification

Dietary intake data were collected at recruitment using a self-reported touchscreen questionnaire (http://biobank.ctsu.ox.ac.uk/showcase/showcase/docs/Touchscreen QuestionsMainFinal.pdf). The frequency of processed meat, beef, lamb, pork, poultry, fish (oily/non-oily), and cheese consumption was coded into three categories: never, less than once a week, and more than once a week. In addition, for vegetables (cooked/raw) and fruit (fresh/dried), consumption was coded into four categories (< 2 servings/d, 2.0–2.9 servings/d, 3.0–3.9 servings/d, ≥ 4 servings/d) as suggested by previous studies [13]. Participants who had missing information or responded with "prefer not to answer" or "do not know" were categorized as "missing". A healthy diet was defined according to the Healthy Eating Score (HDS), which was calculated based on: consuming at least four tablespoons of vegetables each day; consuming at least three pieces of fruit each day; consuming fish at least twice each week; consuming red meat (beef, lamb, and pork) no more than twice each week; consuming processed meat no more than twice each week. One point was awarded for each advantageous dietary factor, with the total diet score ranging from 0 to 5 [14]. Participants were grouped into poor dietary patterns (score 0 or 1), medium dietary patterns (score 2 or 3), and ideal dietary patterns (score 4 or 5) [15].

The frequency of alcohol consumption was collected through a questionnaire, and we dichotomized it into whether or not had daily alcohol drinking. Participants reported different types of alcohol, including beer/cider, white wine/champagne, fortified wine, red wine, spirits, and “other”. According to official UKB statistics, a pint or can of beer/lager/cider = 2 units, a 25 ml single shot of spirits = 1 unit, and a standard glass of wine (175 ml) = 2 units were used to estimate alcohol content. When the alcohol consumption was reported monthly, we divided the intake by 4.3. Then, the weekly intake divided by 7 is the daily unit of consumption. The World Health Organization recommends no more than two "standard intakes of alcohol" per day, and alcohol consumption was therefore dichotomized accordingly to investigate their potential interaction with dietary factors. In the questionnaire, the participants also provided information on whether the alcohol was usually taken together with meals.

### Polygenic risk score

Blood samples were collected from the participants upon enrollment, and genotyped using the UK Biobank Axiom array. A brief description of the procedures for genotype calling, array design, sample handling, quality control, and imputation of the UK Biobank samples has been provided elsewhere [16]. To determine whether the influence of dietary factors varied based on genetic susceptibility to breast cancer, significant SNPs from a recent meta-analysis of breast cancer GWAS were selected to create polygenic risk scores for breast cancer overall and by estrogen receptor (ER) status [11]. For all individuals, the weighted polygenic risk score (PRS) can be used to calculate the PRS, which is the sum of the products of the logarithmic odds ratio (OR) per allele and the allele dose for each SNP associated with breast cancer. Overall, ER+, and ER− PRS were categorized into quartiles, respectively. Detailed information on PRS score generation is provided in Supplementary Table 1.

### Statistical analysis

Cox proportional hazards models were used to assess the associations between dietary factors and breast cancer risk, adjusting for various factors, including smoking status (never, previous, current), ethnicity (grouped into five categories where possible: White, Mixed other, Asian or Asian British, Black or Black British, and unknown), physical activity level (measured in metabolic equivalents task units and categorized into quartiles), Townsend deprivation index (categorized into quintiles), frequency of alcohol intake (≥ once/day or < once/day), employment status (in paid employment, pension, not in paid employment), educational qualifications (college or university degree/vocational qualification; national examination at ages 17–18 years; national examination at age 16 years; other qualifications were treated as missing), body mass index (BMI, categorized as < 18.5, 18.5 to < 25, 25 to < 30, or ≥ 30 kg/m^2^), 22 UKB centers, number of births (categorized as 0, 1, 2, or ≥ 3), age at menarche (categorized as < 13, 13–15, 16 to < 30 years), menopausal status (categorized as no, yes, not sure—had a hysterectomy, or not sure—other reason), age at first birth (categorized as < 23, 23–27, > 27 years, nulliparous/ missing), ever use of oral contraceptive pill (categorized as no or yes), ever use of hormone replacement therapy (categorized as no or yes), and family history of breast cancer (categorized as no or yes). Missingness in the covariates was categorized as a separate category.

To test the multiplicative interaction between the dietary factors and alcohol consumption, as well as between the dietary factors and PRS, an interaction term was included in the regression models and tested using the likelihood ratio (LR) test. Stratified analyses were also conducted according to alcohol intake frequency, whether alcohol was consumed with a meal and quartiles of PRS. We further estimated interaction in the additive scale for dietary factors and PRS using relative excess risk due to interaction (RERI), and a bootstrap approach was used to estimate the confidence interval and the *p* values.

Besides, sensitivity analyses were also performed by stratifying the analyses by menopausal status, Townsend deprivation index, and educational level. Likelihood ratio (LR) tests were used to examine the potential interaction by these variables. In this sensitivity analysis, menopausal status was divided into pre- and post-menopause. The Townsend deprivation index was dichotomized by median, and education was divided into a college degree and others.

All statistical analyses were performed using Stata 15.1. All *P* values were two-sided, and a *P* value of less than 0.05 was considered statistically significant.

## Results

### Baseline characteristics

Among all 273,382 women in the UK Biobank, 58 withdrew their consent and were dropped, and 11,471 women were excluded due to a breast cancer diagnosis before baseline, leaving 261,853 participants in our study with 9069 incident breast cancer cases (Supplementary Fig. 1). The median follow-up time was 10.8 years and the incidence rate was 327.457/100000 person-year in the cohort.

Table 1 shows the characteristics of all participants. Breast cancer cases were more likely to be White ethnicity, less physically active, living in more affluent areas (measured by Townsend score), paid employed, holding a university/college degree/NVQ, consuming more alcohol, smoking, having two or more children, experiencing menarche from 13 to 15 years old, being postmenopausal, having their first birth at an age over 23 years old, taking oral contraceptives or hormone replacement therapy, having no family history of breast cancer, and having a higher BMI.

**Table 1:** Baseline characteristics in the UK Biobank, by frequency of breast cancer (*N* = 261,853)

Characteristic	All participants	Participants who developed
	(*n* = 261,853)	Breast cancer (*n* = 9069)
Means of age at recruitment	59.64 ± 1.53	
Ethnicity, *n* (%)		
White	236,135 (90.18)	8253 (91.00)
Mixed other	9273 (3.54)	309 (3.41)
Asian or British Asian	11,238 (4.29)	374 (4.12)
Black or Black British	1462 (0.56)	32 (0.35)
Unknown	2519 (0.96)	67 (0.74)
Missing	1226 (0.47)	34 (0.37)
Physical activity level, *n* (%)		
Quartile 1	50,535 (19.30)	1834 (20.22)
Quartile 2	50,499 (19.29)	1831 (20.19)
Quartile 3	50,472 (19.27)	1711 (18.87)
Quartile 4	50,492 (19.28)	1584 (17.47)
Unknown	59,855 (22.86)	2109 (23.26)
Townsend deprivation index, *n* (%)		
Quartile 1	52,405 (20.01)	1891 (20.85)
Quartile 2	52,214 (19.94)	1799 (19.84)
Quartile 3	52,312 (19.98)	1803 (19.88)
Quartile 4	52,305 (19.97)	1889 (20.83)
Quartile 5	52,305 (19.97)	1683 (18.56)
Unknown	312 (0.12)	4 (0.04)
Employment, *n* (%)		
In paid employment	144,683 (55.25)	4778 (52.68)
Retired	89,602 (34.22)	3450 (38.04)
Not in paid employment	26,100 (9.97)	795 (8.77)
Unknown	1468 (0.56)	46 (0.51)
Qualification, *n* (%)		
College/university degree/NVQ	113,318 (43.28)	3855 (42.51)
National examination at ages 17–18	36,889 (14.09)	1282 (14.14)
National examination at age 16	93,191 (35.59)	3257 (35.91)
Others/unknown	18,455 (7.05)	675 (7.44)
Body mass index(kg/m^2^), *n* (%)		
< 18.5	1997 (0.76)	57 (0.63)
18.5 to < 25	101,395 (38.72)	3251 (35.85)
25 to < 30	95,394 (36.43)	3396 (37.45)
≥ 30	61,665 (23.55)	2317 (25.55)
Unknown	1402 (0.54)	48 (0.53)
Alcohol intake frequency, *n* (%)		
≥ once/day	41,853 (15.98)	1634 (18.02)
< once/day	219,291 (83.75)	7418 (81.80)
Unknown	709 (0.27)	17 (0.19)
Smoking, *n* (%)		
Never	155,651 (59.44)	5213 (57.48)
Previous	81,280 (31.04)	2966 (32.70)
Current	23,476 (8.97)	852 (9.39)
Unknown	1446 (0.55)	38 (0.42)
Number of births, *n* (%)		
0	48,862 (18.66)	1787 (19.70)
1	34,895 (13.33)	1252 (13.81)
2	114,079 (43.57)	3948 (43.53)
≥ 3	63,216 (24.14)	2060 (22.71)
Unknown	801 (0.31)	22 (0.24)
Age at menarche—years, *n* (%)		
< 13	98,504 (37.62)	3562 (39.28)
13–15	139,793 (53.39)	4724 (52.09)
16 to < 30	15,034 (5.74)	526 (5.80)
Unknown	8522 (3.25)	257 (2.83)
Menopausal status, *n* (%)		
No	63,520 (24.26)	1892 (20.86)
Yes	156,335 (59.7)	5709 (62.95)
Not sure—had a hysterectomy	29,912 (11.42)	1063 (11.72)
Not sure—other reason	11,107 (4.24)	379 (4.18)
Unknown	979 (0.37)	26 (0.29)
Age at frst birth—years, *n* (%)		
< 23	51,487 (19.66)	1696 (18.70)
23–27	71,581 (27.34)	2387 (26.32)
> 27	54,226 (20.71)	1925 (21.23)
Unknown	84,559 (32.29)	3061 (33.75)
Oral contraceptive pill use, *n* (%)		
No	48,881 (18.67)	1759 (19.40)
Yes	211,593 (80.81)	7275 (80.22)
Unknown	1379 (0.53)	35 (0.39)
Hormone replacement therapy, *n* (%)		
No	160,979 (61.48)	5222 (57.58)
Yes	99,350 (37.94)	3800 (41.90)
Unknown	1524 (0.58)	47 (0.52)
Family history, *n* (%)		
No	216,149 (82.55)	7062 (77.87)
Yes	26,537 (10.13)	1365 (15.05)
Unknown	19,167 (7.32)	642 (7.08)

*NVQ* national vocational qualification

### Diet, alcohol consumption, and risk of breast cancer

**Fig. 1:**
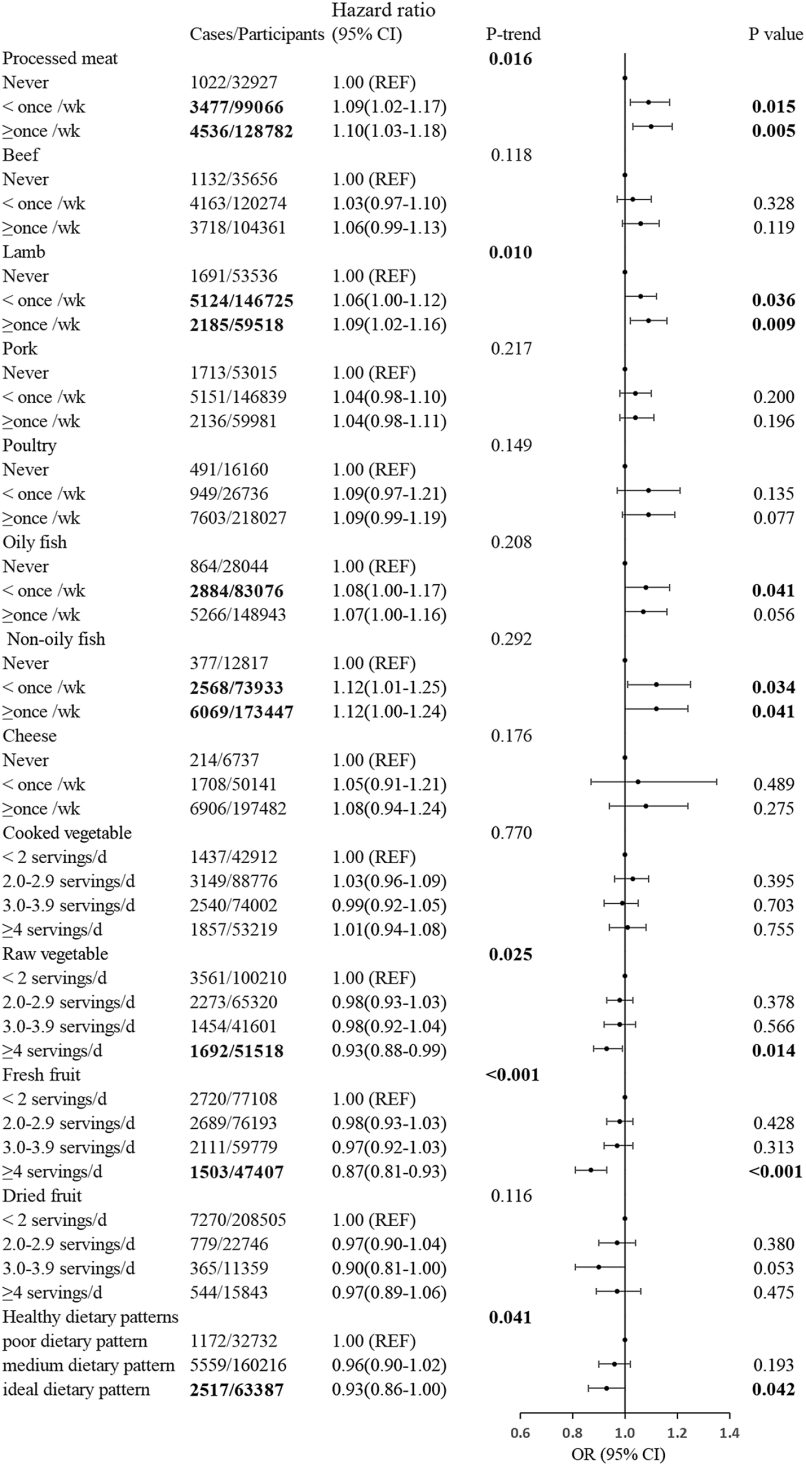
HRs (95% CIs) for the associations between dietary and breast cancer in UK Biobank participants, by frequency of alcohol consumption. Multivariable Cox regression model adjusted for age at recruitment, smoking, ethnicity, physical activity level, Townsend deprivation index, alcohol intake frequency, employment status, educational qualifications, BMI, 22 UKB centers, number of births, age at menarche, menopausal status, age at first birth, ever use of oral contraceptive pill, ever use of hormone replacement therapy, family history of breast cancer, stratified by alcohol intake frequency. The age at the end of the follow-up (i.e., attained age) was used as the underlying time scale. REF, reference

**Fig. 2:**
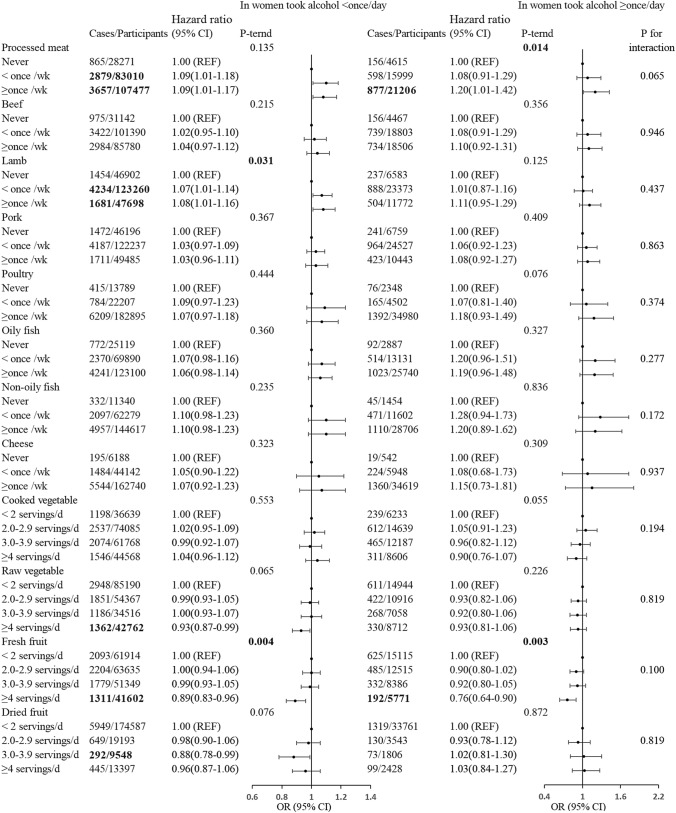
Associations between diet and any breast cancer, by frequency of alcohol consumption (in women took alcohol < 1/d or in women took alcohol ≥ 1/d). Multivariable Cox regression model adjusted for age at recruitment, smoking, ethnicity, physical activity level, Townsend deprivation index, alcohol intake frequency, employment status, educational qualifications, BMI, 22 UKB centers, number of births, age at menarche, menopausal status, age at first birth, ever use of oral contraceptive pill, ever use of hormone replacement therapy, family history of breast cancer, stratified by alcohol intake frequency. The age at the end of the follow-up (i.e., attained age) was used as the underlying time scale. REF,reference

Figure 1 shows the HRs and 95% CIs for breast cancer by consumption level of each food item, using the lowest consumption category as the reference. It is noteworthy that an increase in the risk of breast cancer was observed with the frequent consumption of processed and red meat (HR for processed meat ≥ once/week = 1.10, 95% CI 1.03, 1.18, *P*-trend = 0.016; HR for lamb ≥ once/week = 1.09, 95% CI 1.02–1.16, *P*-trend = 0.010). However, the intake of beef, pork, poultry, cooked vegetables, and cheese was not significantly associated with the risk of breast cancer. Conversely, women with high levels of raw vegetables, fresh fruits, and a healthy dietary pattern may have a reduced risk of breast cancer, with corresponding HRs (95% CIs) of 0.93 (0.88, 0.99), 0.87 (0.81, 0.93), and 0.93 (0.86, 1.00), *P*-trend of 0.025, < 0.001, and 0.041, respectively. Among women who consumed alcohol ≥ once/day, processed meat intake was positively associated with the risk of breast cancer [HR (95% CI)1.20 (1.01, 1.29), *p*-trend = 0.014], while fresh fruit intake was negatively associated with the risk [HR (95% CI) 0.76 (0.64, 0.90), *p*-trend = 0.003] (Fig. 2). Moreover, a borderline significant interaction with alcohol consumption was also observed for processed meat (*P* for interaction = 0.065). To further investigate the synergistic effect between food and alcohol, we stratified the analysis by timing of alcohol consumption (Table 2 and Supplementary Table 2). In women who usually took alcohol together with the meal, the *p* for interaction between processed meat and alcohol consumption was 0.18. In these women, fresh fruit intake was negatively associated with the risk of breast cancer [HR (95% CI):0.73 (0.59–0.91), *p*-trend < 0.01], although the interaction with alcohol consumption was not statistically significant.

**Table 2:** The interaction between meat and alcohol consumption on breast cancer risk, by the timing of alcohol consumption

	Alcohol usually taken with meals (Yes)					Alcohol usually taken with meals (No)				
	Total no	No. of cases	Haz ratio (95% CI)	*P* value	*P*-trend	Total no	No. of cases	Haz ratio (95% CI)	*P* value	*P*-trend
Overall										
°Processed meat					0.29					0.14
Never	11,297	375	1.00 (REF)			9845	299	1.00 (REF)		
< once/wk	38,670	1390	1.05 (0.94–1.18)	0.41		33,443	1152	1.09 (0.96–1.24)	0.19	
≥ once/wk	46,121	1679	1.07 (0.95–1.20)	0.26		48,814	1702	1.11 (0.98–1.26)	0.09	
In women took alcohol < once/day										
°Processed meat					0.96					0.35
Never	8733	285	1.00 (REF)			7794	233	1.00 (REF)		
< once/wk	29,491	1043	1.04 (0.91–1.19)	0.53		26,625	901	1.09 (0.95–1.27)	0.22	
≥ once/wk	34,961	1207	1.02 (0.90–1.17)	0.73		38,773	1297	1.10 (0.95–1.26)	0.20	
In women took alcohol ≥ once/day										
°Processed meat					**0.05**					0.15
Never	2564	90	1.00 (REF)			2051	66	1.00 (REF)		
< once/wk	9179	347	1.07 (0.84–1.35)	0.59		6818	251	1.09 (0.83–1.43)	0.55	
≥ once/wk	11,160	472	1.20 (0.95–1.51)	0.12		10,041	405	1.18 (0.91–1.54)	0.22	
*P* for interaction				0.18					0.51	
Overall										
°Beef					0.66					0.32
Never	10,988	377	1.00 (REF)			11,296	356	1.00 (REF)		
< once/wk	44,747	1595	0.99 (0.88–1.11)	0.83		42,929	1453	1.03 (0.92–1.16)	0.64	
≥ once/wk	40,253	1467	1.01 (0.90–1.13)	0.85		37,698	1336	1.06 (0.94–1.19)	0.36	
In women took alcohol < once/day										
°Beef					0.92					0.45
Never	8676	287	1.00 (REF)			9141	290	1.00 (REF)		
< once/wk	34,425	1193	0.99 (0.87–1.13)	0.93		34,449	1116	0.98 (0.86–1.12)	0.79	
≥ once/wk	30,005	1050	1.00 (0.88–1.14)	0.98		29,445	1019	1.03 (0.90–1.17)	0.69	
In women took alcohol ≥ once/day										
°Beef					0.50					0.58
Never	2312	90	1.00 (REF)			2155	66	1.00 (REF)		
< once/wk	10,322	402	0.96 (0.76–1.21)	0.73		8480	337	1.23 (0.95–1.61)	0.12	
≥ once/wk	10,248	417	1.03 (0.82–1.30)	0.80		8253	317	1.18 (0.90–1.54)	0.24	
*P* for interaction				0.92					0.42	
Overall										
°Lamb					0.15					0.19
Never	15,461	518	1.00 (REF)			19,015	592	1.00 (REF)		
< once/wk	55,820	1967	1.01 (0.91–1.11)	0.87		53,097	1841	1.07 (0.98–1.18)	0.13	
≥ once/wk	24,604	949	1.07 (0.96–1.19)	0.21		19,658	710	1.08 (0.97–1.21)	0.17	
In women took alcohol < once/day										
°Lamb					0.34					0.30
Never	12,294	400	1.00 (REF)			15,600	473	1.00 (REF)		
< once/wk	43,138	1482	1.02 (0.91–1.14)	0.75		42,407	1438	1.09 (0.98–1.21)	0.12	
≥ once/wk	17,596	643	1.06 (0.93–1.20)	0.37		14,899	512	1.07 (0.94–1.22)	0.29	
In women took alcohol ≥ once/day										
°Lamb					0.23					0.40
Never	3167	118	1.00 (REF)			3415	119	1.00 (REF)		
< once/wk	12,682	485	0.97 (0.79–1.19)	0.80		10,690	403	1.03 (0.83–1.26)	0.80	
≥ once/wk	7008	306	1.09 (0.88–1.36)	0.42		4759	198	1.10 (0.87–1.39)	0.42	
*P* for interaction				0.51					0.64	
Overall										
°Pork					0.36					0.79
Never	16,176	545	1.00 (REF)			16,990	561	1.00 (REF)		
< once/wk	56,762	2042	1.02 (0.93–1.13)	0.62		53,281	1823	1.00 (0.91–1.10)	0.98	
≥ once/wk	22,936	851	1.05 (0.94–1.17)	0.37		21,491	760	1.01 (0.91–1.13)	0.80	
In women took alcohol < once/day										
°Pork					0.52					0.94
Never	12,734	415	1.00 (REF)			13,673	450	1.00 (REF)		
< once/wk	43,228	1510	1.03 (0.93–1.15)	0.56		42,289	1391	0.97 (0.87–1.08)	0.54	
≥ once/wk	17,055	604	1.04 (0.92–1.19)	0.50		16,935	584	1.00 (0.88–1.13)	0.99	
In women took alcohol ≥ once/day										
°Pork					0.47					0.71
Never	3442	130	1.00 (REF)			3317	111	1.00 (REF)		
< once/wk	13,534	532	1.00 (0.82–1.21)	0.98		10,992	432	1.14 (0.92–1.40)	0.23	
≥ once/wk	5881	247	1.07 (0.86–1.32)	0.56		4556	176	1.08 (0.84–1.37)	0.55	
*P* for interaction				0.86					0.56	

Multivariable Cox regression model adjusted for age at recruitment, smoking, ethnicity, physical activity level, Townsend deprivation index, alcohol intake frequency, employment status, educational qualifications, BMI, 22 UKB centers, number of births, age at menarche, menopausal status, age at first birth, ever use of oral contraceptive pill, ever use of hormone replacement therapy, family history of breast cancer, stratified by alcohol intake frequency, alcohol, and meal

Stronger associations between fresh fruit, processed meat, and the risk of breast cancer were also observed in women who consumed alcohol ≥ 2 units per day. However, their interactions with a dosage of alcohol consumption were not statistically significant (Supplementary Table 3).

For sensitivity analysis, when stratifying the analysis by menopausal status, processed meat, beef, and lamb were associated with the risk of breast cancer in postmenopausal women, while statistically significant association only persisted in pre-menopausal women who took processed meat ≥ once/week and alcohol ≥ once/day (Supplementary Table 4). Stratification by Townsend deprivation index and education levels suggested no interaction between socio-economic status and those dietary factors identified to be associated with breast cancer in the main analysis, although the point estimates changed slightly (Supplementary Tables 5, 6).

### Diet, genetic predisposition, and risk of breast cancer

**Fig. 3:**
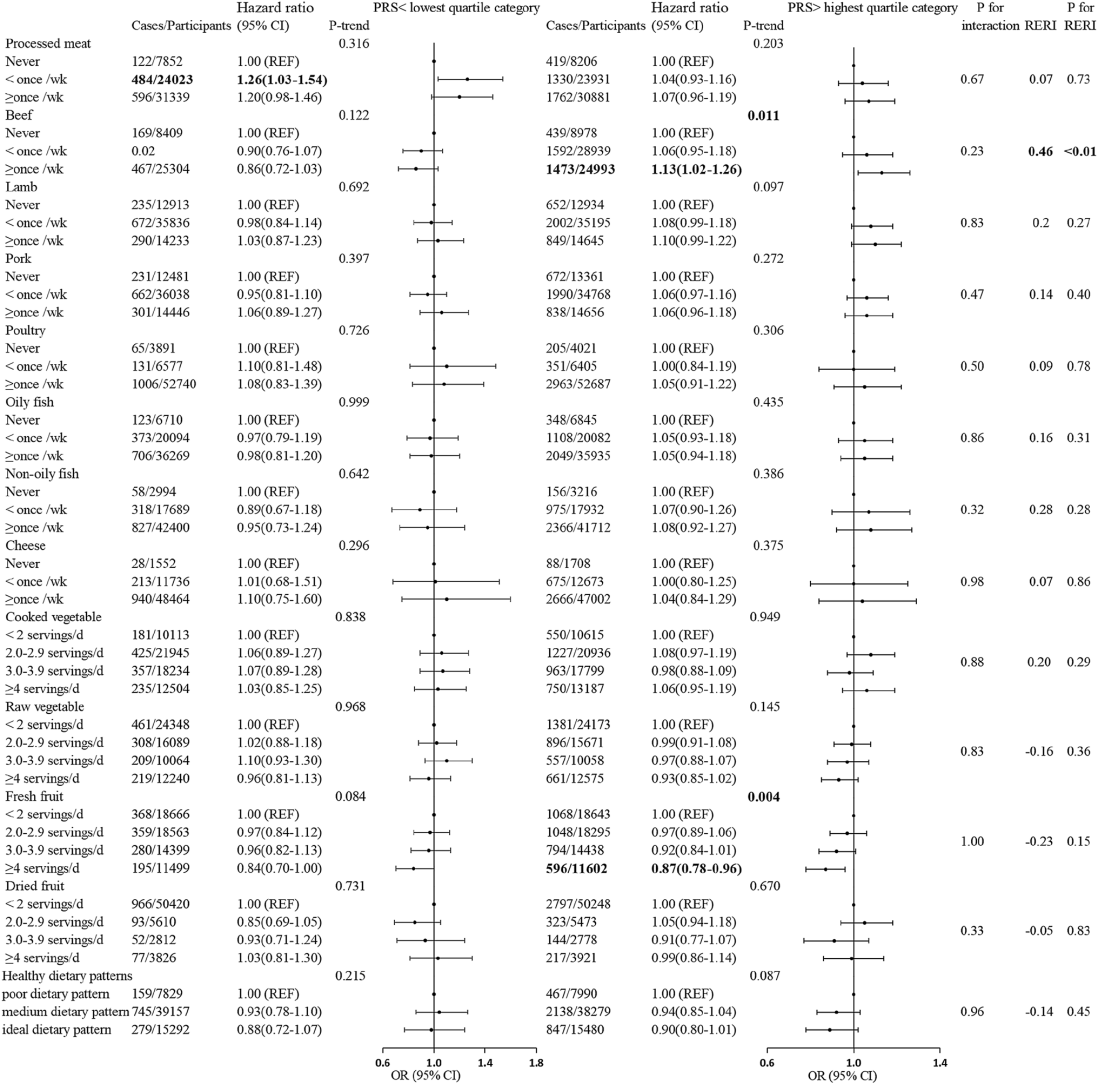
Associations between diet and breast cancer for individuals with different breast cancer PRSs. Multivariable Cox regression model adjusted for age at recruitment, smoking, ethnicity, physical activity level, Townsend deprivation index, alcohol intake frequency, employment status, educational qualifications, BMI, 22 UKB centers, number of births, age at menarche, menopausal status, age at first birth, ever use of oral contraceptive pill, ever use of hormone replacement therapy, family history of breast cancer, stratified by alcohol intake frequency. The age at the end of the follow-up (i.e., attained age) was used as the underlying time scale. REF, reference

The association between dietary factors and breast cancer risk was additionally compared among women with the highest and the lowest quartile of PRS (Fig. 3). In women with the highest quartile of PRS, the HRs (95% CIs) for breast cancer were 1.13 (1.02–1.26) for the highest levels of beef consumption compared with the lowest levels. Although multiplicative interaction was not observed, the RERI of beef consumption in women with the highest quartile of PRS was 0.46, and the *p* value of RERI was < 0.01, which was statistically significant. This significant interaction was still observed when stratified by genetic susceptibility to breast cancer using ER+ PRS and ER− PRS in quartiles (Supplementary Table 7). No interaction was observed for other food.

## Discussion

### Key results

We found a significant association between processed meat, vegetables, fruits, and HDS and the risk of breast cancer, particularly in women with a high frequency of alcohol consumption. Only a borderline significant interaction between processed meat and alcohol consumption was observed among women. We have also discovered a stronger association between beef consumption and breast cancer in women who have a strong genetic predisposition to the disease. No interaction was observed for other dietary factors.

The significant association between processed meat intake and breast cancer risk in our study is supported by several previous findings [8, 17]. Processed meat contains carcinogenic components that can directly damage DNA, such as heterocyclic aromatic amines (HAA) and polycyclic aromatic hydrocarbons (PAHs) resulting from meat processing or preparation, including high-temperature cooking. Nitrites, used as additives, can also induce the formation of *n*-nitroso compounds (NOCs) in the digestive tract, as confirmed in both animal and human biomonitoring studies [18]. These compounds can also play a carcinogenic role through other mechanisms, such as the estrogenic properties of pseudo-hot isostatic pressing (PhIP) [19, 20].

Furthermore, we found a penitential interaction between intake of processed meat and alcohol consumption on the risk of developing breast cancer. Recent large-scale prospective studies have shown that alcohol intake may increase the risk of breast cancer [21–23], likely due to hormonal influences [24–26]. Meanwhile, alcohol has also been suggested to have toxic effects and these effects were mediated by DNA damage and carcinogenic effects of alcohol and through mutagenesis by acetaldehyde and by induction of oxidative damage [27–29]. One plausible mechanism for the synergistic effect between processed meat and alcohol is that alcohol may enhance the penetration of carcinogenic compounds in processed meat as a solvent [30]. For instance, the concurrent consumption of processed meat and alcohol may result in an increased expression of CYP2E1, leading to elevated levels of oxidative stress and DNA damage. Consequently, CYP2E1 reinforced the activation of Reactive Oxygen Species (ROS) that cause DNA damage through ethanol and ROS production. This activation further stimulated PhIP through an oxidation process, which could trigger or sustain tumor growth [26]. Despite these, the synergic effect between processed meat and alcohol still requires more studies to verify.

Consistent with previous studies [6, 9], our findings support the view that an overall healthy dietary pattern was negatively associated with the risk of breast cancer, and this appears to be due to high consumption of vegetables and fruits [31]. Vegetables and fruits are abundant in potentially anti-carcinogenic nutrients including fiber, vitamins C and E, carotenoids, and other bioactive substances [32–34], which may lessen the risk of developing cancer.

In the current study, we observed an association between beef consumption and the risk of breast cancer in women who had the highest quartile of breast cancer PRS. Several studies have also observed additive interactions between lifestyle factors [35], and the genetic predisposition to breast cancer risk [36–38]. Moreover, the slightly higher risk of breast cancer among women with high beef consumption and ER+ PRS compared to the overall PRS may suggest a stronger association with ER-positive disease [39, 40]. Our finding of the additive interaction between ER+ PRS and beef further suggested the role of genetic testing in individualized dietary intervention for breast cancer.

The main strength of our study is its large sample size and population-based cohort design. Other strengths include the UK Biobank cohort’s abundant lifestyle and genetic data, which allowed us to explore the gene and lifestyle interactions for the associations investigated. However, our study has several limitations. On one hand, there is no real measurement of alcohol consumption, but only frequency of alcohol consumption. On the other hand, the data from the touchscreen dietary questionnaire might be affected by recall bias. Besides, as we have performed a lot of interaction tests and subgroup analyses, findings with nominal *P* values for these analyses should be interpreted with caution, considering the multiple testing issues. Further studies are, therefore, needed to validate our findings. Finally, given the observational nature of this study, it is possible that there are still unmeasured confounding factors or residual confounding in our analysis.

In conclusion, our findings support the view that processed meat, vegetable, fresh fruits, and HDS can affect the risk of breast cancer, suggesting that a combined intervention consisting of a well-balanced diet that includes lower processed meat consumption and an increase in vegetable and fresh fruit intake may contribute to preventing breast cancer in women at high risk. However, no strong interaction of dietary factors with alcohol consumption and genetic predisposition was observed for the risk of breast cancer.

### Supplementary Information

Below is the link to the electronic supplementary material.

Supplementary file1 (PDF 1188 KB)

Supplementary file2 (DOCX 112 KB)

## Data Availability

Data from the UK Biobank (http://www.ukbiobank.ac.uk/) are available to researchers upon application. This research was conducted using the UK Biobank Resource under Application 61083.
