# Supplementary material for: The interaction of diet, alcohol, genetic predisposition, and the risk of breast cancer: a cohort study from the UK Biobank
Source: Eur J Nutr. 2023 Nov 1;63(2):343–56. doi: 10.1007/s00394-023-03269-8 (PMC10899287; doi:10.1007/s00394-023-03269-8)
Supplement: Supplementary file 1 — Supplementary file1 (PDF 1188 KB) [file 394_2023_3269_MOESM1_ESM.pdf]

Supplementary Table 1: Detailed information of single nucleotide polymorphisms (SNPs) used for constructing the polygenic risk score (PRS) for breast cancer

SNP[a]	Chr	Position[b]	Reference Allele	Effect Allele	EAF[c]	beta<br>Over BC	beta<br>ER-positive	beta<br>ER-negative	Missing SNPs
1_100880328_A_T	1	100880328	A	T	0.4097	0.0373	0.0373	0.0373	
1_10566215_A_G	1	10566215	A	G	0.3290	-0.0586	-0.0407	-0.1109	
1_110198129_CAAA	1	110198129	CAAA	C	0.7755	0.0458	0.0458	0.0458	
1_114445880_G_A	1	114445880	G	A	0.1664	0.0621	0.0621	0.0621	
1_118141492_A_C	1	118141492	A	C	0.2657	0.0452	0.0452	0.0452	
1_120257110_T_C	1	120257110	T	C	0.5309	0.0385	0.0430	0.0226	
1_121280613_A_G	1	121280613	A	G	0.4053	0.0881	0.1052	0.0209	
1_121287994_A_G	1	121287994	A	G	0.1060	-0.0673	-0.0814	-0.0114	
1_145604302_C_CT	1	145604302	C	CT	0.3515	-0.0399	-0.0469	-0.0126	
1_149906413_T_C	1	149906413	T	C	0.4017	0.0548	0.0548	0.0548	
1_155556971_G_A	1	155556971	G	A	0.2302	0.0499	0.0499	0.0499	
1_168171052_CA_C	1	168171052	CA	C	0.1097	-0.0680	-0.0680	-0.0680	
1_172328767_T_TA	1	172328767	T	TA	0.3305	-0.0435	-0.0435	-0.0435	
1_18807339_T_C	1	18807339	T	C	0.5145	-0.0564	-0.0649	-0.0248	
1_201437832_C_T	1	201437832	C	T	0.0559	0.0917	0.0917	0.0917	
1_202184600_C_T	1	202184600	C	T	0.3992	-0.0065	0.0133	-0.0822	
1_203770448_T_A	1	203770448	T	A	0.2715	0.0498	0.0498	0.0498	
1_204502514_T_TT(	1	204502514	T	TTCTG/	0.8028	-0.0321	-0.0024	-0.1345	
1_208076291_G_A	1	208076291	G	A	0.3337	-0.0366	-0.0366	-0.0366	
1_217053815_T_G	1	217053815	T	G	0.3280	0.0417	0.0417	0.0417	
1_217220574_G_A	1	217220574	G	A	0.2107	-0.0440	-0.0459	0.0029	
1_220671050_C_T	1	220671050	C	T	0.2415	0.0418	0.0418	0.0418	
1_242034263_A_G	1	242034263	A	G	0.0305	0.1428	0.1428	0.1428	
1_41380440_C_T	1	41380440	C	T	0.6438	0.0426	0.0426	0.0426	
1_41389220_T_C	1	41389220	T	C	0.0169	0.1550	0.1550	0.1550	
1_46670206_TC_T	1	46670206	TC	T	0.2973	0.0447	0.0595	0.0216	
1_51467096_CT_C	1	51467096	CT	C	0.4800	0.0374	0.0374	0.0374	
1_7917076_G_A	1	7917076	G	A	0.3899	-0.0409	-0.0409	-0.0409	
1_88156923_G_A	1	88156923	G	A	0.1487	0.0494	0.0580	0.0183	
1_88428199_C_A	1	88428199	C	A	0.2477	-0.0387	-0.0387	-0.0387	
2_10138983_T_C	2	10138983	T	C	0.1160	0.0603	0.0603	0.0603	
2_121058254_A_G	2	121058254	A	G	0.7047	-0.0334	-0.0232	-0.0682	
2_121089731_T_C	2	121089731	T	C	0.1943	-0.0427	-0.0290	-0.1027	
2_121159205_G_A	2	121159205	G	A	0.3527	-0.0440	-0.0440	-0.0440	
2_121246568_T_C	2	121246568	T	C	0.8970	0.0992	0.0992	0.0992	
2_172974566_C_G	2	172974566	C	G	0.4743	-0.0473	-0.0611	-0.0061	
2_174212910_A_G	2	174212910	A	G	0.8450	0.0593	0.0621	0.0175	
2_192381934_C_T	2	192381934	C	T	0.8588	0.0316	0.0180	0.1012	
2_19315675_T_A	2	19315675	T	A	0.5599	-0.0331	-0.0229	-0.0570	
2_202204741_T_C	2	202204741	T	C	0.7210	-0.0492	-0.0492	-0.0492	
2_217920769_G_T	2	217920769	G	T	0.5001	-0.1318	-0.1532	-0.0589	
2_217955896_GA_G	2	217955896	GA	G	0.0382	-0.2016	-0.2362	-0.0558	
2_218292158_C_G	2	218292158	C	G	0.7309	-0.0757	-0.0757	-0.0757	
2_218714845_G_A	2	218714845	G	A	0.3917	-0.0431	-0.0463	-0.0184	
2_241388857_C_A	2	241388857	C	A	0.9772	-0.1232	-0.1232	-0.1232	
2_25129473_A_G	2	25129473	A	G	0.4082	-0.0427	-0.0427	-0.0427	
2_29179452_G_C	2	29179452	G	C	0.2287	-0.0066	0.0207	-0.1006	

2_29615233_T_C	2	29615233	T	C	0.2622	-0.0427	-0.0427	-0.0427	
2_39699510_C_CT	2	39699510	C	CT	0.4659	-0.0402	-0.0402	-0.0402	
2_70172587_G_A	2	70172587	G	A	0.2787	-0.0412	-0.0412	-0.0412	
2_88358825_G_C	2	88358825	G	C	0.3081	0.0473	0.0473	0.0473	
3_141112859_CTT_(	3	141112859	CTT	C	0.4149	0.0551	0.0551	0.0551	
3_172285237_G_A	3	172285237	G	A	0.2131	0.0422	0.0501	-0.0133	
3_189774456_C_T	3	189774456	C	T	0.2235	-0.0478	-0.0478	-0.0478	
3_27353716_C_A	3	27353716	C	A	0.5259	0.0748	0.0822	0.0310	
3_27388664_C_G	3	27388664	C	G	0.2735	0.0502	0.0502	0.0502	
3_29294845_C_T	3	29294845	C	T	0.0163	-0.1281	-0.1221	-0.2988	
3_30684907_C_T	3	30684907	C	T	0.2975	0.0592	0.0657	0.0170	
3_46888198_T_C	3	46888198	T	C	0.1032	-0.0806	-0.0806	-0.0806	
3_4742251_A_G	3	4742251	A	G	0.3802	0.0616	0.0616	0.0616	
3_49709912_C_CT	3	49709912	C	CT	0.2873	-0.0367	-0.0355	-0.0721	
3_55970777_A_AT	3	55970777	A	AT	0.0305	-0.1195	-0.1195	-0.1195	
3_59373745_C_T	3	59373745	C	T	0.4294	-0.0394	-0.0394	-0.0394	
3_63887449_T_TTG	3	63887449	T	TTG	0.1297	0.0648	0.0648	0.0648	missing
3_71620370_T_G	3	71620370	T	G	0.6382	-0.0374	-0.0374	-0.0374	
3_87037543_A_G	3	87037543	A	G	0.0921	-0.0723	-0.0723	-0.0723	
3_99403877_G_A	3	99403877	G	A	0.4852	-0.0376	-0.0376	-0.0376	
4_106069013_G_T	4	106069013	G	T	0.2289	0.0471	0.0594	0.0097	
4_126752992_A_AA	4	126752992	A	AAT	0.5167	-0.0377	-0.0377	-0.0377	missing
4_143467195_C_T	4	143467195	C	T	0.1115	-0.0569	-0.0569	-0.0569	
4_151218296_CATA	4	151218296	CATATTT	C	0.6533	0.0388	0.0388	0.0388	
4_175842495_G_A	4	175842495	G	A	0.1161	-0.0898	-0.1162	0.0199	
4_175847436_C_A	4	175847436	C	A	0.3433	0.0348	0.0537	-0.0099	
4_187503758_A_T	4	187503758	A	T	0.4471	0.0357	0.0357	0.0357	missing
4_38784633_G_T	4	38784633	G	T	0.2493	0.0489	0.0489	0.0489	
4_84370124_TAA_T	4	84370124	TAA	TA	0.5324	-0.0464	-0.0464	-0.0464	
4_89240476_G_A	4	89240476	G	A	0.4395	0.0352	0.0352	0.0352	
4_92594859_TTCTT	4	92594859	TTCTTTC	T	0.4445	-0.0407	-0.0407	-0.0407	
5_104300273_G_T	5	104300273	G	T	0.1810	-0.0487	-0.0487	-0.0487	
5_122478676_C_A	5	122478676	C	A	0.7448	-0.0386	-0.0386	-0.0386	
5_122705244_C_T	5	122705244	C	T	0.0306	0.0944	0.0944	0.0944	
5_1279790_C_T	5	1279790	C	T	0.2592	0.0617	0.0325	0.1502	
5_1296255_A_AG	5	1296255	A	AG	0.3072	-0.0549	-0.0417	-0.1056	
5_131640536_A_G	5	131640536	A	G	0.5427	0.0392	0.0467	0.0099	
5_132407058_C_T	5	132407058	C	T	0.2450	-0.0388	-0.0561	-0.0214	
5_1353077_T_C	5	1353077	T	C	0.0121	0.1552	0.1552	0.1552	
5_158244083_C_T	5	158244083	C	T	0.5683	-0.0677	-0.0677	-0.0677	
5_16231194_G_C	5	16231194	G	C	0.5594	-0.0426	-0.0426	-0.0426	
5_169591460_T_C	5	169591460	T	C	0.3345	0.0412	0.0501	0.0182	
5_173358154_G_A	5	173358154	G	A	0.4074	0.0365	0.0365	0.0365	
5_176134882_T_C	5	176134882	T	C	0.5422	0.0363	0.0363	0.0363	
5_2777029_G_A	5	2777029	G	A	0.4139	0.0391	0.0391	0.0391	
5_32579616_TCA_T	5	32579616	TCA	T	0.4844	0.0363	0.0363	0.0363	
5_345109_T_C	5	345109	T	C	0.0544	0.0840	0.0840	0.0840	missing
5_44508264_G_GT	5	44508264	G	GT	0.1265	-0.1177	-0.1177	-0.1177	
5_44619502_A_G	5	44619502	A	G	0.1549	-0.1101	-0.1101	-0.1101	
5_44649944_C_T	5	44649944	C	T	0.6010	0.0492	0.0713	-0.0261	
5_44706498_A_G	5	44706498	A	G	0.2481	0.0497	0.0648	-0.0256	
5_44853593_G_C	5	44853593	G	C	0.3081	-0.0336	-0.0222	-0.0778	

5_52679539_C_CA	5	52679539	C	CA	0.0998	0.0571	0.0571	0.0571	missing
5_55662540_C_CT	5	55662540	C	CT	0.3631	-0.0458	-0.0458	-0.0458	
5_55965167_C_T	5	55965167	C	T	0.5576	0.0394	0.0394	0.0394	
5_56023083_T_G	5	56023083	T	G	0.1583	0.1366	0.1612	0.0686	
5_56042972_C_T	5	56042972	C	T	0.0521	0.0865	0.1082	0.0058	
5_56045081_T_C	5	56045081	T	C	0.1655	-0.0564	-0.0643	-0.0168	
5_58241712_C_T	5	58241712	C	T	0.5750	-0.0434	-0.0434	-0.0434	
5_71965007_G_A	5	71965007	G	A	0.2572	-0.0410	-0.0410	-0.0410	
5_73234583_T_C	5	73234583	T	C	0.3213	-0.0363	-0.0494	-0.0101	
5_77155397_GT_G	5	77155397	GT	G	0.3466	-0.0408	-0.0408	-0.0408	missing
5_79180995_G_GA	5	79180995	G	GA	0.1755	0.0328	0.0248	0.0804	
5_81512947_TA_T	5	81512947	TA	T	0.2503	-0.0598	-0.0731	-0.0342	
5_90789470_G_A	5	90789470	G	A	0.1580	-0.0564	-0.0714	-0.0031	
6_130341728_C_CT	6	130341728	C	CT	0.7116	0.0472	0.0472	0.0472	
6_13713366_G_C	6	13713366	G	C	0.5691	-0.0553	-0.0623	-0.0152	
6_149595505_T_C	6	149595505	T	C	0.2061	-0.0476	-0.0476	-0.0476	
6_151949806_A_C	6	151949806	A	C	0.3083	0.0703	0.0541	0.1103	
6_151955914_A_G	6	151955914	A	G	0.0713	0.1449	0.1150	0.2240	
6_152022664_CAAA	6	152022664	CAAAAAA	C	0.6119	0.0137	0.0137	0.0137	
6_152023191_G_A	6	152023191	G	A	0.3965	0.0626	0.0509	0.1008	
6_152055978_A_T	6	152055978	A	T	0.0627	0.0740	0.0740	0.0740	
6_152432902_C_T	6	152432902	C	T	0.5146	0.0649	0.0527	0.0965	
6_16399557_C_T	6	16399557	C	T	0.3299	-0.0373	-0.0373	-0.0373	
6_169006947_C_G	6	169006947	C	G	0.5202	-0.0308	-0.0252	-0.0628	
6_170332621_T_C	6	170332621	T	C	0.6158	0.0373	0.0373	0.0373	
6_18783140_G_A	6	18783140	G	A	0.6200	0.0326	0.0478	0.0033	
6_20537845_CA_C	6	20537845	CA	C	0.4733	-0.0391	-0.0391	-0.0391	
6_21923810_T_C	6	21923810	T	C	0.4303	-0.0321	-0.0438	-0.0032	
6_27425644_G_C	6	27425644	G	C	0.0815	-0.0737	-0.0737	-0.0737	
6_43227141_G_A	6	43227141	G	A	0.0985	-0.0640	-0.0640	-0.0640	
6_82263549_AAT_A	6	82263549	AAT	A	0.4262	0.0477	0.0477	0.0477	
6_85912194_CAA_C	6	85912194	CAA	C	0.0604	0.0762	0.0762	0.0762	
6_87803819_T_C	6	87803819	T	C	0.2770	0.0383	0.0318	0.0678	
7_101552440_G_A	7	101552440	G	A	0.1255	-0.0568	-0.0568	-0.0568	
7_102481842_T_C	7	102481842	T	C	0.3416	0.0418	0.0418	0.0418	
7_130656911_C_T	7	130656911	C	T	0.3734	-0.0476	-0.0476	-0.0476	
7_130674481_G_A	7	130674481	G	A	0.2971	0.0416	0.0416	0.0416	
7_139943702_CT_C	7	139943702	CT	C	0.5381	0.0582	0.0666	0.0057	
7_144048902_G_T	7	144048902	G	T	0.2284	-0.0563	-0.0592	-0.0148	
7_21940960_A_G	7	21940960	A	G	0.3515	-0.0467	-0.0467	-0.0467	
7_25569548_C_T	7	25569548	C	T	0.1667	-0.0486	-0.0486	-0.0486	
7_28869017_G_A	7	28869017	G	A	0.1072	-0.0572	-0.0572	-0.0572	
7_55192256_A_C	7	55192256	A	C	0.5497	-0.0349	-0.0349	-0.0349	
7_91459189_A_ATT	7	91459189	A	ATT	0.3286	0.0452	0.0452	0.0452	missing
7_94113799_T_C	7	94113799	T	C	0.2792	0.0449	0.0449	0.0449	
7_98005235_G_A	7	98005235	G	A	0.1627	-0.0467	-0.0467	-0.0467	
7_99948655_T_G	7	99948655	T	G	0.2109	0.0420	0.0420	0.0420	
8_102483100_T_C	8	102483100	T	C	0.0967	0.0593	0.0736	0.0137	
8_106358620_A_T	8	106358620	A	T	0.1003	-0.0745	-0.0895	-0.0100	
8_117209548_A_G	8	117209548	A	G	0.6445	-0.0417	-0.0417	-0.0417	
8_120862186_A_G	8	120862186	A	G	0.1318	0.0527	0.0527	0.0527	
8_124563705_T_C	8	124563705	T	C	0.1458	0.0477	0.0477	0.0477	

8_124571581_G_A	8	124571581	G	A	0.4173	0.0340	0.0340	0.0340
8_124739913_T_G	8	124739913	T	G	0.3985	0.0466	0.0466	0.0466
8_128213561_C_CA	8	128213561	C	CA	0.4153	-0.0430	-0.0430	-0.0430
8_128370949_C_G	8	128370949	C	G	0.4020	0.0642	0.0820	0.0076
8_128372172_A_G	8	128372172	A	G	0.5446	0.0597	0.0597	0.0597
8_129199566_G_A	8	129199566	G	A	0.1717	0.0615	0.0615	0.0615
8_143669254_A_G	8	143669254	A	G	0.3390	-0.0346	-0.0346	-0.0346
8_170692_T_C	8	170692	T	C	0.2227	0.0477	0.0348	0.1040
8_17787610_CT_C	8	17787610	CT	C	0.6230	-0.0377	-0.0377	-0.0377
8_23447496_A_G	8	23447496	A	G	0.6487	-0.0389	-0.0389	-0.0389
8_23663653_C_A	8	23663653	C	A	0.4032	0.0335	0.0451	0.0059
8_29509616_A_C	8	29509616	A	C	0.6756	-0.0601	-0.0601	-0.0601
8_36858483_A_G	8	36858483	A	G	0.1820	-0.0760	-0.0760	-0.0760
8_76230943_A_G	8	76230943	A	G	0.8282	0.0755	0.0755	0.0755
8_76333056_C_T	8	76333056	C	T	0.0878	0.1129	0.1129	0.1129
8_76378165_G_T	8	76378165	G	T	0.3595	-0.0391	-0.0391	-0.0391
9_110303808_TAA_	9	110303808	TAA	T	0.2065	0.0797	0.1007	0.0130
9_110837073_A_G	9	110837073	A	G	0.0630	0.1158	0.1315	0.0289
9_110837176_C_T	9	110837176	C	T	0.1750	0.0653	0.0809	-0.0037
9_110849525_G_T	9	110849525	G	T	0.5977	0.0153	0.0153	0.0153
9_110885479_C_T	9	110885479	C	T	0.6222	0.0877	0.1110	0.0019
9_119313486_A_G	9	119313486	A	G	0.4087	-0.0462	-0.0462	-0.0462
9_129424719_A_G	9	129424719	A	G	0.4577	-0.0382	-0.0382	-0.0382
9_136146597_C_T	9	136146597	C	T	0.2727	0.0400	0.0400	0.0400
9_21964882_CAAAA	9	21964882	CAAAA	C	0.3184	0.0550	0.0550	0.0550
9_22041998_C_G	9	22041998	C	G	0.1393	0.0289	0.0168	0.0906
9_36928288_T_C	9	36928288	T	C	0.5349	0.0249	0.0249	0.0249
9_6880263_A_G	9	6880263	A	G	0.2855	0.0348	0.0499	-0.0078
9_87782211_T_C	9	87782211	T	C	0.5094	0.0361	0.0361	0.0361
9_98362587_T_C	9	98362587	T	C	0.0940	0.0576	0.0576	0.0576
10_114777670_C_T	10	114777670	C	T	0.4631	0.0472	0.0472	0.0472
10_115128491_T_C	10	115128491	T	C	0.7846	-0.0592	-0.0592	-0.0592
10_123095209_G_A	10	123095209	G	A	0.3269	-0.0538	-0.0702	0.0048
10_123340107_A_G	10	123340107	A	G	0.0656	0.1508	0.1837	0.0053
10_123340431_GC_	10	123340431	GC	G	0.5963	-0.2408	-0.2913	-0.0326
10_123349324_A_T	10	123349324	A	T	0.0484	-0.2609	-0.3270	-0.0137
10_13892298_G_A	10	13892298	G	A	0.4376	0.0371	0.0371	0.0371
10_22032942_A_G	10	22032942	A	G	0.7085	-0.0580	-0.0719	0.0344
10_22477776_ACC_	10	22477776	ACC	A	0.0202	0.1687	0.1687	0.1687
10_22861490_A_C	10	22861490	A	C	0.9370	0.0875	0.0960	0.0201
10_38523626_C_A	10	38523626	C	A	0.3698	0.0404	0.0404	0.0404
10_5794652_A_G	10	5794652	A	G	0.2137	0.0470	0.0470	0.0470
10_64299890_A_G	10	64299890	A	G	0.1603	-0.1345	-0.1428	-0.1030
10_64819996_G_T	10	64819996	G	T	0.1958	0.0472	0.0472	0.0472
10_71335574_C_T	10	71335574	C	T	0.3179	-0.0404	-0.0404	-0.0404
10_80851257_G_T	10	80851257	G	T	0.6172	-0.0805	-0.0898	-0.0443
10_80886726_A_G	10	80886726	A	G	0.1631	0.0762	0.0762	0.0762
10_95292187_CAA_	10	95292187	CAA	C	0.8234	-0.0512	-0.0512	-0.0512
11_103614438_T_G	11	103614438	T	G	0.6572	0.0147	0.0029	0.0676
11_108267402_C_C	11	108267402	C	CA	0.4173	-0.0022	0.0141	-0.0629
11_111696440_T_C	11	111696440	T	C	0.6221	-0.0396	-0.0396	-0.0396
11_116727936_A_T	11	116727936	A	T	0.2046	-0.0423	-0.0423	-0.0423

11_122966626_A_G	11	122966626	A	G	0.2922	-0.0383	-0.0383	-0.0383
11_129243417_T_G	11	129243417	T	G	0.8620	-0.0543	-0.0543	-0.0543
11_129461016_A_G	11	129461016	A	G	0.6016	0.0453	0.0453	0.0453
11_18664241_T_G	11	18664241	T	G	0.7293	0.0461	0.0461	0.0461
11_1895708_C_A	11	1895708	C	A	0.3924	-0.0762	-0.0762	-0.0762
11_42844441_C_T	11	42844441	C	T	0.3279	-0.0336	-0.0336	-0.0336
11_433617_T_C	11	433617	T	C	0.7969	-0.0437	-0.0437	-0.0437
11_44368892_G_A	11	44368892	G	A	0.5495	0.0374	0.0374	0.0374
11_46318032_C_G	11	46318032	C	G	0.0659	-0.0748	-0.0748	-0.0748
11_65553492_C_A	11	65553492	C	A	0.1867	0.0425	0.0425	0.0425
11_65572431_G_A	11	65572431	G	A	0.4886	-0.0347	-0.0448	-0.0067
11_69328130_A_T	11	69328130	A	T	0.2130	-0.0423	-0.0538	0.0143
11_69330983_G_A	11	69330983	G	A	0.1250	0.1022	0.1240	0.0174
11_69331418_C_T	11	69331418	C	T	0.0753	0.1782	0.2018	0.0066
11_803017_A_G	11	803017	A	G	0.5167	0.0457	0.0457	0.0457
12_103097887_C_T	12	103097887	C	T	0.1175	0.0546	0.0611	0.0149
12_111600134_G_T	12	111600134	G	T	0.3715	-0.0442	-0.0442	-0.0442
12_115108136_T_C	12	115108136	T	C	0.2615	0.0465	0.0465	0.0465
12_115796577_A_G	12	115796577	A	G	0.1959	-0.0428	-0.0643	-0.0148
12_115835836_T_C	12	115835836	T	C	0.4171	-0.0813	-0.0977	-0.0153
12_120832146_C_T	12	120832146	C	T	0.1593	0.0516	0.0516	0.0516
12_14413931_G_C	12	14413931	G	C	0.2619	0.0484	0.0484	0.0484
12_28149568_C_T	12	28149568	C	T	0.1170	-0.0620	-0.0620	-0.0620
12_28174817_C_T	12	28174817	C	T	0.2421	-0.0856	-0.0856	-0.0856
12_28347382_C_T	12	28347382	C	T	0.2153	-0.0521	-0.0521	-0.0521
12_29140260_G_A	12	29140260	G	A	0.9126	0.0647	0.0647	0.0647
12_293626_A_G	12	293626	A	G	0.3711	0.0401	0.0401	0.0401
12_57146069_T_G	12	57146069	T	G	0.1037	-0.0579	-0.0579	-0.0579
12_70798355_A_T	12	70798355	A	T	0.1810	0.0469	0.0469	0.0469
12_83064195_G_GA	12	83064195	G	GA	0.0992	0.0671	0.0671	0.0671
12_85004551_C_T	12	85004551	C	T	0.4955	0.0348	0.0348	0.0348
12_96027759_A_G	12	96027759	A	G	0.2963	-0.0867	-0.0867	-0.0867
13_32839990_G_A	13	32839990	G	A	0.0174	0.0424	0.0424	0.0424
13_32972626_A_T	13	32972626	A	T	0.0079	0.2687	0.2308	0.4284
13_43501356_A_G	13	43501356	A	G	0.8303	0.0517	0.0458	0.0975
13_73806982_T_C	13	73806982	T	C	0.3153	0.0345	0.0251	0.0653
13_73960952_A_G	13	73960952	A	G	0.7618	0.0399	0.0368	0.0730
14_105213978_T_G	14	105213978	T	G	0.4588	0.0399	0.0399	0.0399
14_37128564_C_A	14	37128564	C	A	0.2122	-0.0733	-0.0850	-0.0339
14_37228504_C_T	14	37228504	C	T	0.4434	0.0390	0.0390	0.0390
14_68660428_T_C	14	68660428	T	C	0.8345	-0.0474	-0.0612	0.0245
14_68979835_T_C	14	68979835	T	C	0.2581	-0.0911	-0.0911	-0.0911
14_91751788_TC_T	14	91751788	TC	T	0.6934	0.0380	0.0447	0.0091
14_91841069_A_G	14	91841069	A	G	0.3444	0.0513	0.0513	0.0513
14_93070286_C_T	14	93070286	C	T	0.1709	-0.0577	-0.0577	-0.0577
15_100905819_A_C	15	100905819	A	C	0.1100	-0.0608	-0.0608	-0.0608
15_46680811_C_A	15	46680811	C	A	0.0115	-0.1973	-0.1973	-0.1973
15_50694306_A_G	15	50694306	A	G	0.3446	-0.0417	-0.0417	-0.0417
15_66630569_G_A	15	66630569	G	A	0.6413	-0.0369	-0.0369	-0.0369
15_67457698_A_G	15	67457698	A	G	0.0496	0.0782	0.0990	0.0141
15_75750383_T_C	15	75750383	T	C	0.2604	-0.0413	-0.0413	-0.0413
15_91512267_G_T	15	91512267	G	T	0.1353	-0.0589	-0.0589	-0.0589

16_10706580_G_A	16	10706580	G	A	0.0695	-0.0740	-0.0740	-0.0740
16_23007047_G_T	16	23007047	G	T	0.0236	0.1218	0.1218	0.1218
16_4008542_CAAAA	16	4008542	CAAAAA	C	0.8213	-0.0329	-0.0184	-0.0892
16_4106788_C_A	16	4106788	C	A	0.2643	-0.0300	-0.0182	-0.0782
16_52538825_C_A	16	52538825	C	A	0.2562	0.1147	0.1147	0.1147
16_52599188_C_T	16	52599188	C	T	0.2406	0.1070	0.1070	0.1070
16_53809123_C_T	16	53809123	C	T	0.4201	-0.0704	-0.0651	-0.0957
16_53861139_C_T	16	53861139	C	T	0.7604	-0.0338	-0.0167	-0.0782
16_53861592_G_A	16	53861592	G	A	0.3663	-0.0337	-0.0337	-0.0337
16_54682064_G_A	16	54682064	G	A	0.4850	0.0477	0.0477	0.0477
16_6963972_C_G	16	6963972	C	G	0.7835	0.0354	0.0303	0.0811
16_80648296_A_G	16	80648296	A	G	0.2303	0.0839	0.0890	0.0467
16_85145977_T_C	16	85145977	T	C	0.4856	-0.0211	-0.0044	-0.0714
16_87086492_T_C	16	87086492	T	C	0.2586	-0.0469	-0.0469	-0.0469
17_29168077_G_T	17	29168077	G	T	0.2613	-0.0568	-0.0568	-0.0568
17_39251123_T_C	17	39251123	T	C	0.0682	0.0799	0.0631	0.1431
17_40127060_T_C	17	40127060	T	C	0.0570	0.0174	-0.0161	0.1511
17_40485239_G_T	17	40485239	G	T	0.0874	-0.0571	-0.0416	-0.1142
17_40744470_G_A	17	40744470	G	A	0.0124	0.2017	0.2017	0.2017
17_43212339_C_CT	17	43212339	C	CT	0.2284	0.0438	0.0438	0.0438
17_44283858_G_A	17	44283858	G	A	0.1895	-0.0540	-0.0540	-0.0540
17_53209774_A_C	17	53209774	A	C	0.3023	-0.0793	-0.0933	-0.0365
17_77781725_A_G	17	77781725	A	G	0.5038	-0.0401	-0.0401	-0.0401
18_11696613_C_T	18	11696613	C	T	0.1379	-0.0381	-0.0281	-0.0940
18_20634253_C_T	18	20634253	C	T	0.6403	-0.0415	-0.0415	-0.0415
18_24125857_T_C	18	24125857	T	C	0.4214	0.0346	0.0346	0.0346
18_24337424_C_G	18	24337424	C	G	0.6205	0.0455	0.0455	0.0455
18_24518050_AT_A	18	24518050	AT	A	0.2773	-0.0599	-0.0830	0.0060
18_25407513_C_G	18	25407513	C	G	0.7126	0.0399	0.0307	0.0648
18_29981526_G_A	18	29981526	G	A	0.0474	-0.1058	-0.1058	-0.1058
18_42411803_G_C	18	42411803	G	C	0.0717	-0.0877	-0.1037	-0.0189
18_42888797_T_C	18	42888797	T	C	0.3519	-0.0542	-0.0542	-0.0542
19_13249921_G_T	19	13249921	G	T	0.0513	0.0956	0.0956	0.0956
19_17393925_C_A	19	17393925	C	A	0.2958	0.0378	0.0036	0.1692
19_18569492_C_T	19	18569492	C	T	0.3481	-0.0719	-0.0719	-0.0719
19_19517054_C_CG	19	19517054	C	CGGGC	0.3537	0.0437	0.0437	0.0437
19_44283031_T_C	19	44283031	T	C	0.3519	0.0619	0.0619	0.0619
19_46166073_T_C	19	46166073	T	C	0.6074	-0.0360	-0.0447	-0.0117
19_55816678_C_T	19	55816678	C	T	0.3626	-0.0359	-0.0359	-0.0359
20_11379842_T_C	20	11379842	T	C	0.9483	0.0844	0.0844	0.0844
20_41613706_C_G	20	41613706	C	G	0.7928	0.0315	0.0266	0.0784
20_52296849_G_A	20	52296849	G	A	0.2400	0.0440	0.0539	0.0144
20_5948227_G_A	20	5948227	G	A	0.0628	0.0760	0.0760	0.0760
21_16364756_T_G	21	16364756	T	G	0.1732	0.0646	0.0646	0.0646
21_16566350_A_G	21	16566350	A	G	0.0873	0.0595	0.0678	0.0172
21_16574455_C_A	21	16574455	C	A	0.3167	-0.0707	-0.0808	-0.0329
21_47762932_G_A	21	47762932	G	A	0.0355	0.0946	0.0946	0.0946
22_19766137_C_T	22	19766137	C	T	0.3798	-0.0367	-0.0367	-0.0367
22_29121087_A_G	22	29121087	A	G	0.0054	0.1839	0.2812	-0.1566
22_29135543_G_A	22	29135543	G	A	0.0870	0.0654	0.0654	0.0654
22_29203724_C_T	22	29203724	C	T	0.0209	0.1405	0.1793	0.0191
22_29551872_A_G	22	29551872	A	G	0.9846	-0.1716	-0.1716	-0.1716

22_38583315_AAAA	22	38583315	AAAAG	AAAAG	0.2805	-0.0471	-0.0608	0.0079	missing
22_39343916_T_A	22	39343916	T	A	0.2541	0.0407	0.0407	0.0407	
22_40904707_CT_C	22	40904707	CT	C	0.1099	0.1148	0.1148	0.1148	
22_43433100_C_T	22	43433100	C	T	0.1144	-0.0600	-0.0600	-0.0600	
22_45319953_G_A	22	45319953	G	A	0.4166	-0.0134	-0.0060	-0.0611	
22_46283297_G_A	22	46283297	G	A	0.1117	0.0736	0.0736	0.0736	

[b]Position based on build 37

[c]EAF, Effect Allele frequency estimates for controls in the complete Oncoarray dataset

Taken from: Mavaddat et al.Polygenic Risk Scores for Prediction ofBreast Cancer and Breast Cancer Subtypes. Am JHum Genet.2019; 104(1): 21-34

Supplementary Table 2. The interaction between dietary factors and alcohol consumption on breast cancer risk, by timing of alcohol consumption.

	**Alcohol usually taken with meals (Yes)**			**Alcohol usually taken with meals (No)**		
	Haz Ratio (95%CI)	P value	P-trend	Haz Ratio (95%CI)	P value	P-trend
Overall						
cooked vegetable			0.22			0.75
< 2 servings/d	1.00 (REF)			1.00 (REF)		
2.0-2.9 servings/d	1.02(0.91-1.13)	0.79		1.11(1.00-1.23)	0.05	
3.0-3.9 servings/d	0.96(0.86-1.08)	0.50		1.05(0.94-1.17)	0.39	
≥4 servings/d	0.96(0.85-1.08)	0.50		1.06(0.94-1.19)	0.35	
In women took alcohol<once/day						
cooked vegetable			0.81			0.76
< 2 servings/d	1.00 (REF)			1.00 (REF)		
2.0-2.9 servings/d	1.02(0.89-1.16)	0.78		1.11(0.99-1.25)	0.08	
3.0-3.9 servings/d	1.00(0.88-1.14)	1.00		1.05(0.92-1.18)	0.48	
≥4 servings/d	1.03(0.89-1.19)	0.68		1.06(0.93-1.22)	0.83	
In women took alcohol≥once/day						
cooked vegetable			**<0.01**			0.86
< 2 servings/d	1.00 (REF)			1.00 (REF)		
2.0-2.9 servings/d	1.00(0.81-1.24)	0.97		1.09(0.88-1.36)	0.42	
3.0-3.9 servings/d	0.87(0.70-1.08)	0.20		1.06(0.85-1.34)	0.60	
≥4 servings/d	0.79(0.62-1.00)	**0.04**		1.05(0.82-1.34)	0.71	
P for interaction		0.15			0.52	
Overall						
Raw vegetable			0.32			0.73
< 2 servings/d	1.00 (REF)			1.00 (REF)		
2.0-2.9 servings/d	1.02(0.94-1.11)	0.65		1.01(0.92-1.10)	0.82	
3.0-3.9 servings/d	1.00(0.90-1.10)	0.62		1.02(0.92-1.13)	0.72	
≥4 servings/d	0.95(0.86-1.05)	0.30		0.97(0.88-1.08)	0.60	
In women took alcohol<once/day						
Raw vegetable			0.80			0.80
< 2 servings/d	1.00 (REF)			1.00 (REF)		
2.0-2.9 servings/d	1.06(0.96-1.17)	0.22		1.03(0.93-1.14)	0.62	
3.0-3.9 servings/d	1.07(0.96-1.20)	0.22		1.00(0.89-1.13)	0.98	
≥4 servings/d	0.97(0.86-1.08)	0.55		0.99(0.88-1.10)	0.80	
In women took alcohol≥once/day						
Raw vegetable			0.12			0.88
< 2 servings/d	1.00 (REF)			1.00 (REF)		
2.0-2.9 servings/d	0.91(0.77-1.07)	0.27		0.96(0.80-1.16)	0.68	
3.0-3.9 servings/d	0.80(0.66-0.98)	**0.03**		1.07(0.87-1.33)	0.51	
≥4 servings/d	0.90(0.75-1.08)	0.27		0.95(0.77-1.17)	0.62	
P for interaction		0.16			0.87	
Overall						
Fresh fruit			**<0.01**			0.19
< 2 servings/d	1.00 (REF)			1.00 (REF)		
2.0-2.9 servings/d	0.98(0.90-1.07)	0.62		0.96(0.88-1.05)	0.41	
3.0-3.9 servings/d	0.93(0.85-1.02)	0.14		1.01(0.92-1.11)	0.83	
≥4 servings/d	0.85(0.77-0.95)	**<0.01**		0.90(0.81-1.01)	0.06	
In women took alcohol<once/day						
Fresh fruit			0.07			0.47
< 2 servings/d	1.00 (REF)			1.00 (REF)		
2.0-2.9 servings/d	1.04(0.94-1.15)	0.47		0.96(0.86-1.06)	0.42	
3.0-3.9 servings/d	0.97(0.87-1.09)	0.62		1.01(0.91-1.13)	0.83	
≥4 servings/d	0.91(0.80-1.03)	0.12		0.93(0.82-1.05)	0.26	
In women took alcohol≥once/day						
Fresh fruit			**<0.01**			0.17

< 2 servings/d	1.00 (REF)			1.00 (REF)		
2.0-2.9 servings/d	0.84(0.71-0.99)	**0.03**		0.98(0.82-1.18)	0.85	
3.0-3.9 servings/d	0.85(0.71-1.01)	0.07		1.01(0.82-1.25)	0.90	
≥4 servings/d	0.73(0.59-0.91)	**0.01**		0.78(0.60-1.01)	0.06	
P for interaction		0.27			0.41	
Overall						
Dried fruit			0.93			**0.04**
< 2 servings/d	1.00 (REF)			1.00 (REF)		
2.0-2.9 servings/d	1.01(0.91-1.13)	0.81		0.96(0.84-1.10)	0.53	
3.0-3.9 servings/d	0.95(0.82-1.11)	0.54		0.78(0.64-0.97)	**0.02**	
≥4 servings/d	1.01(0.88-1.15)	0.89		0.91(0.77-1.08)	0.29	
In women took alcohol<once/day						
Dried fruit			0.95			**0.01**
< 2 servings/d	1.00 (REF)			1.00 (REF)		
2.0-2.9 servings/d	1.08(0.95-1.23)	0.23		0.92(0.78-1.07)	0.27	
3.0-3.9 servings/d	0.93(0.77-1.12)	0.43		0.72(0.56-0.92)	**0.01**	
≥4 servings/d	1.01(0.87-1.18)	0.89		0.87(0.71-1.05)	0.15	
In women took alcohol≥once/day						
Dried fruit			0.83			0.51
< 2 servings/d	1.00 (REF)			1.00 (REF)		
2.0-2.9 servings/d	0.83(0.65-1.05)	0.12		1.11(0.84-1.47)	0.45	
3.0-3.9 servings/d	1.02(0.76-1.37)	0.89		1.03(0.68-1.55)	0.90	
≥4 servings/d	1.01(0.88-1.31)	0.94		1.09(0.78-1.53)	0.62	
P for interaction		0.46			0.37	
Overall						
Oily fish			0.54			0.47
Never	1.00 (REF)			1.00 (REF)		
< once /wk	1.19(1.01-1.39)	**0.03**		1.01(0.89-1.15)	0.85	
⩾once /wk	1.15(0.98-1.33)	0.08		1.04(0.92-1.17)	0.56	
In women took alcohol<once/day						
Oily fish			0.46			0.93
Never	1.00 (REF)			1.00 (REF)		
< once /wk	1.17(0.98-1.39)	0.09		0.98(0.86-1.13)	0.81	
⩾once /wk	1.14(0.96-1.35)	0.13		1.00(0.87-1.14)	0.96	
In women took alcohol≥once/day						
Oily fish			1.00			0.19
Never	1.00 (REF)			1.00 (REF)		
< once /wk	1.26(0.89-1.77)	0.18		1.16(0.86-1.56)	0.33	
⩾once /wk	1.18(0.85-1.64)	0.33		1.22(0.92-1.63)	0.17	
P for interaction		0.10			0.74	
Overall						
Non-oily fish			0.47			0.91
Never	1.00 (REF)			1.00 (REF)		
< once /wk	0.99(0.81-1.21)	0.93		1.17(0.97-1.42)	0.11	
⩾once /wk	1.02(0.84-1.24)	0.82		1.12(0.93-1.35)	0.24	
In women took alcohol<once/day						
Non-oily fish			0.26			0.80
Never	1.00 (REF)			1.00 (REF)		
< once /wk	0.95(0.76-1.20)	0.68		1.11(0.89-1.37)	0.35	
⩾once /wk	1.02(0.82-1.27)	0.88		1.06(0.87-1.31)	0.55	
In women took alcohol≥once/day						
Non-oily fish			0.66			0.87
Never	1.00 (REF)			1.00 (REF)		
< once /wk	1.10(0.72-1.67)	0.66		1.47(0.94-2.31)	0.09	
⩾once /wk	1.04(0.69-1.57)	0.84		1.37(0.88-2.12)	0.16	
P for interaction		0.36			0.22	

Overall						
Poultry			1.00			0.74
Never	1.00 (REF)			1.00 (REF)		
< once /wk	0.99(0.83-1.19)	0.93		1.16(0.96-1.40)	0.13	
⩾once /wk	1.00(0.85-1.16)	0.96		1.09(0.93-1.28)	0.30	
In women took alcohol<once/day						
Poultry			0.35			0.95
Never	1.00 (REF)			1.00 (REF)		
< once /wk	1.01(0.82-1.25)	0.90		1.15(0.93-1.43)	0.20	
⩾once /wk	0.95(0.80-1.14)	0.58		1.06(0.88-1.27)	0.52	
In women took alcohol≥once/day						
Poultry			0.12			0.41
Never	1.00 (REF)			1.00 (REF)		
< once /wk	0.93(0.63-1.35)	0.69		1.21(0.82-1.79)	0.34	
⩾once /wk	1.14(0.82-1.57)	0.44		1.20(0.85-1.68)	0.30	
P for interaction		0.15			0.35	
Overall						
Cheese			**0.04**			0.50
Never	1.00 (REF)			1.00 (REF)		
< once /wk	1.17(0.86-1.59)	0.31		0.83(0.65-1.06)	0.13	
⩾once /wk	1.26(0.93-1.69)	0.13		0.84(0.67-1.06)	0.13	
In women took alcohol<once/day						
Cheese			**0.04**			0.20
Never	1.00 (REF)			1.00 (REF)		
< once /wk	1.31(0.91-1.89)	0.14		0.76(0.59-0.98)	**0.04**	
⩾once /wk	1.41(0.99-2.01)	0.06		0.76(0.60-0.97)	**0.03**	
In women took alcohol≥once/day						
Cheese			0.59			0.32
Never	1.00 (REF)			1.00 (REF)		
< once /wk	0.83(0.47-1.47)	0.52		1.61(0.71-3.67)	0.26	
⩾once /wk	0.90(0.52-1.57)	0.72		1.68(0.75-3.75)	0.21	
P for interaction		0.73			0.12	

MultivariableCox regression model adjusted for age at recruitment, smoking, ethnicity, physical activity level, BMI, townsend deprivation index, alcohol intake frequency, employment status, educational qualifications, 22 UKB centers, number of births, age at menarche, menopausal status, age at first birth, ever use of oral contraceptive pill use, ever use of hormone replacement therapy, family history of breast cancer, stratified by alcohol intake frequency,

Supplementary Table 3. The association between dietary factors and breast cancer risk, by alcohol intake unites.

	alcohol<2 units/day				alcohol≥2 units/day				P for interaction
	Cases/Participants	Hazard ratio(95% CI)	P-ternd	P value	Cases/Participants	Hazard ratio(95% CI)	P-ternd	P value	
Processed meat			0.199				**0.028**		0.235
Never	759/24729	1.00 (REF)			262/8157	1.00 (REF)			
< once /wk	2359/69751	1.06(0.98-1.16)		0.135	**1118/29258**	**1.16(1.01-1.32)**		**0.036**	
≥once /wk	2988/89018	1.07(0.98-1.16)		0.112	**1546/39665**	**1.19(1.04-1.36)**		**0.011**	
Beef			0.267				0.481		0.878
Never	851/27497	1.00 (REF)			280/8112	1.00 (REF)			
< once /wk	2839/85743	1.02(0.94-1.10)		0.705	1322/34450	1.06(0.93-1.21)		0.356	
≥once /wk	2395/69833	1.04(0.96-1.13)		0.326	1323/34453	1.07(0.93-1.21)		0.347	
Lamb			**0.006**				0.762		0.596
Never	1237/40676	1.00 (REF)			454/12809	1.00 (REF)			
< once /wk	3443/102683	1.07(1.00-1.14)		0.051	1679/43950	1.03(0.92-1.14)		0.631	
≥once /wk	**1395/39305**	**1.12(1.03-1.21)**		**0.005**	790/20165	1.02(0.91-1.15)		0.709	
Pork			0.129				0.694		0.543
Never	1253/40221	1.00 (REF)			460/12734	1.00 (REF)			
< once /wk	3395/101344	1.03(0.96-1.10)		0.406	1756/45420	1.03(0.93-1.14)		0.549	
≥once /wk	1429/41173	1.06(0.98-1.15)		0.135	705/18755	0.99(0.88-1.11)		0.827	
Poultry			0.234				0.550		0.741
Never	351/11918	1.00 (REF)			140/4219	1.00 (REF)			
< once /wk	669/19633	1.08(0.95-1.23)		0.255	280/7076	1.11(0.9-1.36)		0.32	
≥once /wk	5093/152074	1.08(0.97-1.21)		0.165	2508/65801	1.09(0.91-1.29)		0.351	
Oily fish			0.510				0.389		0.376
Never	671/22176	1.00 (REF)			193/5830				
< once /wk	1982/59022	1.07(0.98-1.17)		0.113	902/23999	1.10(0.94-1.29)		0.231	
≥once /wk	3436/101710	1.05(0.97-1.15)		0.221	1828/47130	1.10(0.95-1.28)		0.212	
Non-oily fish			0.228				0.758		0.067
Never	300/10178	1.00 (REF)			77/2616	1.00 (REF)			
< once /wk	1751/53268	1.06(0.94-1.20)		0.326	**817/20613**	**1.31(1.03-1.65)**		**0.026**	
≥once /wk	4042/119562	1.08(0.96-1.22)		0.201	2025/53761	1.22(0.97-1.53)		0.091	
Cheese			0.537				0.192		0.884
Never	180/5720	1.00 (REF)			34/1010	1.00 (REF)			

< once /wk	1265/38090	1.04(0.89-1.21)		0.659	443/12000	1.11(0.78-1.57)		0.572	
≥once /wk	4502/134522	1.05(0.90-1.22)		0.554	2402/62837	1.17(0.83-1.64)		0.372	
Cooked vegetable			0.742				0.813		0.336
< 2 servings/d	1030/31297	1.00 (REF)			407/11575	1.00 (REF)			
2.0-2.9 servings/d	2092/62177	1.00(0.92-1.07)		0.933	1057/26547	1.09(0.97-1.23)		0.126	
3.0-3.9 servings/d	1717/51412	0.99(0.91-1.07)		0.722	822/22543	0.99(0.87-1.11)		0.807	
≥4 servings/d	1229/37073	0.99(0.91-1.08)		0.801	628/16101	1.05(0.93-1.2)		0.422	
Raw vegetable			**0.011**				0.590		0.638
< 2 servings/d	2554/73566	1.00 (REF)			1005/26568	1.00 (REF)			
2.0-2.9 servings/d	1490/45005	0.96(0.90-1.02)		0.206	783/20278	1.01(0.92-1.11)		0.867	
3.0-3.9 servings/d	930/28303	0.95(0.88-1.03)		0.2	524/13271	1.03(0.93-1.15)		0.528	
≥4 servings/d	**1086/34848**	**0.91(0.85-0.98)**		**0.012**	606/16626	0.96(0.87-1.06)		0.414	
Fresh fruit			**0.001**				**0.039**		0.235
< 2 servings/d	1724/51260	1.00 (REF)			994/25769	1.00 (REF)			
2.0-2.9 servings/d	1814/53007	0.99(0.93-1.06)		0.774	875/23143	0.96(0.87-1.05)		0.348	
3.0-3.9 servings/d	1452/43189	0.96(0.89-1.03)		0.253	659/16546	1.00(0.90-1.10)		0.992	
≥4 servings/d	**1107/35814**	**0.88(0.81-0.95)**		**0.001**	**396/11559**	**0.85(0.75-0.95)**		**0.006**	
Dried fruit			0.092				0.850		0.692
< 2 servings/d	4877/145405	1.00 (REF)			2391/62943	1.00 (REF)			
2.0-2.9 servings/d	548/16465	0.98(0.89-1.07)		0.608	231/6271	0.95(0.83-1.09)		0.449	
3.0-3.9 servings/d	248/8173	0.89(0.78-1.01)		0.065	117/3181	0.94(0.78-1.13)		0.498	
≥4 servings/d	376/11625	0.95(0.85-1.05)		0.328	168/4200	1.03(0.88-1.20)		0.742	
Healthy dietary patterns			**0.022**				0.691		0.257
poor dietary pattern	792/22272	1.00 (REF)			380/10428	1.00 (REF)			
medium dietary pattern	**3726/112673**	**0.92(0.85-0.99)**		**0.027**	1831/47452	1.04(0.94-1.17)		0.41	
ideal dietary pattern	**11462/44744**	**0.89(0.82-0.98)**		**0.013**	695/18607	0.99(0.88-1.13)		0.928	

Supplementary Table 4. The association between processed and red meat and breast cancer risk, by menopausal status.

	In premenopausal women					In postmenopausal women				
	Total No.	No. of cases	Haz Ratio (95%CI)	P value	P-trend	Total No.	No. of cases	Haz Ratio (95%CI)	P value	P-trend
Overall										
Processed meat					0.18					**0.03**
Never	8 325	214	1.00 (REF)			19 950	640	1.00 (REF)		
< once /wk	22 036	**691**	1.22(1.04-1.42)	**0.01**		61 062	**2 248**	1.10(1.01-1.21)	**0.03**	
≥once /wk	33 008	**982**	1.17(1.01-1.37)	**0.04**		74 998	**2 811**	1.12(1.03-1.22)	**0.01**	
In women took										
Processed meat					0.38					0.17
Never	7 290	185	1.00 (REF)			16 915	541	1.00 (REF)		
< once /wk	19 129	**597**	1.23(1.05-1.46)	**0.01**		50 210	1 817	1.09(0.99-1.20)	0.09	
≥once /wk	28 320	809	1.16(0.98-1.36)	0.08		61 488	2 234	1.09(0.99-1.20)	0.08	
In women took alcohol≥once/day										
Processed meat					0.15					**0.03**
Never	1 028	29	1.00 (REF)			3 018	99	1.00 (REF)		
< once /wk	2 896	94	1.10(0.72-1.68)	0.65		10 816	431	1.19(0.96-1.49)	0.11	
≥once /wk	4 666	172	1.28(0.86-1.90)	0.23		13 456	**577**	1.28(1.03-1.58)	**0.03**	
P for interaction				0.07					0.25	
Overall										
Beef					0.22					**0.04**
Never	10 217	272	1.00 (REF)			20 395	666	1.00 (REF)		
< once /wk	28 305	862	1.12(0.97-1.28)	0.11		72 760	2 635	1.05(0.97-1.15)	0.22	
≥once /wk	24 752	753	1.12(0.97-1.29)	0.13		62 562	**2 383**	1.09(1.00-1.19)	**0.04**	
In women took										
Beef					0.38					0.08
Never	9 114	245	1.00 (REF)			17 546	567	1.00 (REF)		
< once /wk	24 585	725	1.08(0.93-1.25)	0.31		60 233	2 125	1.04(0.95-1.14)	0.43	
≥once /wk	20 947	621	1.08(0.93-1.26)	0.30		50 570	1 886	1.08(0.98-1.19)	0.16	
In women took alcohol≥once/day										
Beef					0.30					0.31

Never	1 090	27	1.00 (REF)			2 830	99	1.00 (REF)		
< once /wk	3 706	136	1.42(0.94-2.16)	0.10		12 479	510	1.14(0.91-1.41)	0.25	
≥once /wk	3 788	132	1.38(0.91-2.11)	0.13		11 951	497	1.15(0.92-1.43)	0.21	
P for interaction				0.35					0.88	
Overall										
Lamb					**0.04**					**0.01**
Never	15 774	420	1.00 (REF)			29 699	981	1.00 (REF)		
< once /wk	35 677	**1 103**	1.14(1.02-1.28)	**0.02**		87 716	**3 218**	1.08(1.00-1.16)	**0.04**	
≥once /wk	11 717	**359**	1.15(1.00-1.33)	**0.04**		37 993	**1 478**	1.12(1.03-1.21)	**<0.01**	
In women took										
Lamb					**0.04**					0.06
Never	14 001	362	1.00 (REF)			25 705	839	1.00 (REF)		
< once /wk	30 793	939	1.17(1.04-1.32)	**0.01**		72 472	2 619	1.08(1.00-1.17)	0.06	
≥once /wk	9 759	286	1.16(0.99-1.36)	0.06		29 897	**1 112**	1.10(1.00-1.20)	**0.04**	
In women took alcohol≥once/day										
Lamb					0.62					**0.04**
Never	1 761	58	1.00 (REF)			3 970	142	1.00 (REF)		
< once /wk	4 867	163	0.97(0.71-1.31)	0.83		15 192	599	1.07(0.89-1.28)	0.49	
≥once /wk	1 946	73	1.08(0.76-1.54)	0.66		8 069	366	1.20(0.98-1.46)	0.07	
P for interaction				0.39					0.50	
Overall										
Pork					0.13					0.48
Never	15 322	426	1.00 (REF)			29 727	1 019	1.00 (REF)		
< once /wk	35 227	1 067	1.06(0.94-1.18)	0.35		88 476	3 272	1.04(0.97-1.11)	0.31	
≥once /wk	12 650	390	1.11(0.97-1.28)	0.13		37 211	1 387	1.03(0.95-1.12)	0.43	
In women took										
Pork					0.27					0.65
Never	13 546	376	1.00 (REF)			25 567	869	1.00 (REF)		
< once /wk	30 210	888	1.02(0.91-1.16)	0.64		72 346	2 607	1.02(0.95-1.11)	0.55	
≥once /wk	10 823	324	1.09(0.94-1.27)	0.26		30 175	1 096	1.02(0.93-1.12)	0.63	
In women took alcohol≥once/day										
Pork					0.26					0.50

Never	1 762	50	1.00 (REF)		4 131	150	1.00 (REF)	
< once /wk	5 004	179	1.22(0.89-1.68)	0.22	16 090	665	1.10(0.92-1.32)	0.28
≥once /wk	1 813	65	1.25(0.86-1.81)	0.25	7 006	291	1.09(0.90-1.34)	0.38
P for interaction				0.71				0.76

Multivariable Cox regression model adjusted for age at recruitment, cooked vegetables, fresh fruit intake, smoking, ethnicity, physical activity level, Townsend deprivation index, alcohol intake frequency, employment status, educational qualifications, BMI, 22 UKB centers, number of births, age at menarche, menopausal status, age at first birth, ever use of oral contraceptive pill use, ever use of hormone replacement therapy, family history of breast cancer, stratified by alcohol intake frequency and menopausal status.

Supplementary Table 5. The interaction between dietary factors and alcohol consumption on breast cancer risk, by Townsend deprivation index

	Townsend deprivation index lower category					**Townsend deprivation index higher category**					P for
	Total No.	No. of cases	Haz Ratio (95%CI)	P value	P-trend	Total No.	No. of cases	Haz Ratio (95%CI)	P value	P-trend	interaction
Processed meat					0.21					**0.03**	0.43
Never	14 654	475	1.00 (REF)			18 235	547	1.00 (REF)			
< once /wk	50 797	1 822	1.08(0.97-1.19)	0.15		48 161	1 653	1.10(1.00-1.21)	0.06		
≥once /wk	65 079	2 327	1.08(0.98-1.20)	0.12		63 539	2 207	1.12(1.02-1.24)	**0.02**		
Beef					0.28					0.25	0.99
Never	14 735	481	1.00 (REF)			20 880	651	1.00 (REF)			
< once /wk	59 851	2 115	1.04(0.94-1.15)	0.45		60 276	2 044	1.03(0.94-1.12)	0.58		
≥once /wk	55 796	2 019	1.06(0.96-1.17)	0.27		48 443	1 699	1.05(0.96-1.16)	0.26		
Lamb					0.05					0.08	0.86
Never	24 269	783	1.00 (REF)			29 190	907	1.00 (REF)			
< once /wk	76 105	2 694	1.06(0.98-1.15)	0.18		70 442	2 427	1.07(0.99-1.15)	0.10		
≥once /wk	29 814	1 130	1.10(1.00-1.20)	**0.04**		29 650	1 055	1.09(0.99-1.19)	0.07		
Pork					0.61					**0.03**	0.15
Never	22 825	786	1.00 (REF)			30 117	926	1.00 (REF)			
< once /wk	76 485	2 737	1.01(0.93-1.09)	0.85		70 192	2 412	1.06(0.98-1.14)	0.15		
≥once /wk	30 867	1 088	0.98(0.89-1.08)	0.67		29 039	1 047	1.11(1.01-1.21)	**0.03**		
Poultry					0.56					0.19	0.31
Never	6 743	204	1.00 (REF)			9 398	287	1.00 (REF)			
< once /wk	11 723	449	1.18(1.00-1.40)	**0.04**		14 984	500	1.01(0.88-1.17)	0.86		
≥once /wk	112 087	3 972	1.11(0.97-1.28)	0.14		105 678	3 627	1.07(0.94-1.20)	0.31		
Oily fish					0.78					0.14	0.20
Never	11 228	371	1.00 (REF)			16 775	493	1.00 (REF)			
< once /wk	41 277	1 449	1.03(0.92-1.15)	0.64		41 696	1 434	1.13(1.02-1.25)	**0.02**		
≥once /wk	77 810	2 794	1.03(0.92-1.14)	0.65		70 969	2 469	1.11(1.01-1.23)	**0.04**		
Non-oily fish					0.43					0.51	0.14
Never	5 081	163	1.00 (REF)			7 717	214	1.00 (REF)			
< once /wk	35 274	1 231	1.05(0.89-1.23)	0.60		38 570	1 336	1.19(1.03-1.37)	**0.02**		
≥once /wk	89 991	3 215	1.06(0.91-1.24)	0.46		83 254	2 851	1.15(1.00-1.33)	**0.04**		
Cheese					0.04					0.98	0.32

Never	2 770	78	1.00 (REF)			3 958	134	1.00 (REF)			
< once /wk	23 687	822	1.22(0.97-1.54)	0.09		26 379	886	0.97(0.81-1.16)	0.74		
≥once /wk	101 375	3 621	1.27(1.02-1.60)	**0.03**		95 892	3 283	0.98(0.82-1.17)	0.81		
Cooked vegetable					0.50					0.79	0.61
< 2 servings/d	18 626	654	1.00 (REF)			24 224	783	1.00 (REF)			
2.0-2.9 servings/d	46 577	1 676	1.00(0.92-1.10)	0.92		42 088	1 471	1.05(0.96-1.14)	0.29		
3.0-3.9 servings/d	38 934	1 348	0.96(0.87-1.05)	0.38		34 989	1 191	1.02(0.93-1.11)	0.75		
≥4 servings/d	25 832	924	0.99(0.89-1.09)	0.82		27 333	932	1.03(0.94-1.14)	0.51		
Raw vegetable					0.34					**0.03**	0.86
< 2 servings/d	50 038	1 800	1.00 (REF)			50 053	1 759	1.00 (REF)			
2.0-2.9 servings/d	33 920	1 197	0.98(0.91-1.05)	0.58		31 323	1 075	0.97(0.90-1.05)	0.50		
3.0-3.9 servings/d	21 094	756	1.00(0.91-1.08)	0.91		20 460	698	0.97(0.89-1.06)	0.49		
≥4 servings/d	24 665	847	0.95(0.88-1.04)	0.26		26 789	844	0.91(0.83-0.98)	**0.02**		
Fresh fruit					**<0.01**					**<0.01**	0.29
< 2 servings/d	36 171	1 308	1.00 (REF)			40 830	1 410	1.00 (REF)			
2.0-2.9 servings/d	39 359	1 422	0.99(0.91-1.06)	0.70		36 756	1 266	0.97(0.90-1.05)	0.46		
3.0-3.9 servings/d	31 229	1 134	0.99(0.91-1.07)	0.72		28 483	977	0.96(0.88-1.04)	0.30		
≥4 servings/d	23 672	753	0.85(0.78-0.94)	**<0.01**		23 676	749	0.88(0.81-0.97)	**0.01**		
Dried fruit					0.13					0.47	0.23
< 2 servings/d	103 691	3 717	1.00 (REF)			104 544	3 551	1.00 (REF)			
2.0-2.9 servings/d	11 943	404	0.93(0.84-1.03)	0.16		10 790	375	1.01(0.91-1.13)	0.83		
3.0-3.9 servings/d	5 832	176	0.82(0.71-0.96)	**0.01**		5 521	188	0.99(0.85-1.15)	0.88		
≥4 servings/d	8 071	294	0.99(0.88-1.12)	0.88		7 754	249	0.94(0.83-1.07)	0.35		
Healthy dietary patterns					0.13					0.16	0.47
poor dietary pattern	16 085	584	1.00 (REF)			16 596	588	1.00 (REF)			
medium dietary pattern	81 099	2 882	0.97(0.89-1.06)	0.56		78 923	2 675	0.94(0.86-1.03)	0.20		
ideal dietary pattern	31 949	1 101	0.93(0.84-1.03)	0.17		31 372	1 054	0.92(0.83-1.02)	0.13		

Supplementary Table 6. The interaction between dietary factors and alcohol consumption on breast cancer risk, by education level

	**Holding a college/post-secondary degree /NVQ**					**Educational level lower category**					P for interaction
	Total No.	No. of cases	Haz Ratio (95%CI)	P value	P-trend	Total No.	No. of cases	Haz Ratio (95%CI)	P value	P-trend	
Processed meat					**0.02**					0.28	0.35
Never	16 819	505	1.00 (REF)			16 108	517	1.00 (REF)			
< once /wk	43 666	1 501	1.11(1.00-1.22)	**0.05**		55 400	1 976	1.08(0.98-1.19)	0.13		
≥once /wk	52 675	1 846	1.14(1.03-1.26)	**0.01**		76 107	2 690	1.08(0.98-1.18)	0.13		
Beef					0.08					0.60	0.83
Never	17 883	554	1.00 (REF)			17 773	578	1.00 (REF)			
< once /wk	52 185	1 774	1.04(0.95-1.15)	0.42		68 089	2 389	1.03(0.94-1.13)	0.57		
≥once /wk	42 973	1 517	1.09(0.98-1.20)	0.10		61 388	2 201	1.03(0.94-1.13)	0.52		
Lamb					**0.03**					0.13	0.80
Never	23 785	725	1.00 (REF)			29 751	966	1.00 (REF)			
< once /wk	64 553	2 229	1.08(1.00-1.18)	0.06		82 172	2 895	1.05(0.97-1.13)	0.24		
≥once /wk	24 587	894	1.12(1.01-1.24)	**0.03**		34 931	1 291	1.07(0.98-1.16)	0.12		
Pork					0.30					0.45	0.89
Never	24 748	782	1.00 (REF)			28 267	931	1.00 (REF)			
< once /wk	64 977	2 244	1.04(0.95-1.12)	0.41		81 862	2 907	1.04(0.96-1.12)	0.31		
≥once /wk	23 211	823	1.05(0.95-1.16)	0.30		36 770	1 313	1.04(0.95-1.13)	0.39		
Poultry					0.08					0.76	0.50
Never	9 247	277	1.00 (REF)			6 913	214	1.00 (REF)			
< once /wk	12 654	428	1.05(0.90-1.22)	0.56		14 082	521	1.12(0.96-1.32)	0.15		
≥once /wk	91 307	3 150	1.1(0.97-1.25)	0.13		126 720	4 453	1.08(0.94-1.24)	0.29		
Oily fish					0.89					0.13	0.09
Never	10 644	316	1.00 (REF)			17 400	548	1.00 (REF)			
< once /wk	36 815	1 293	1.13(1.00-1.28)	**0.04**		46 261	1 591	1.05(0.95-1.16)	0.35		
≥once /wk	65 522	2 241	1.08(0.96-1.22)	0.22		83 421	3 025	1.07(0.98-1.18)	0.13		
Non-oily fish					0.97					0.17	0.18
Never	5 967	178	1.00 (REF)			6 850	199	1.00 (REF)			
< once /wk	33 073	1 151	1.11(0.95-1.3)	0.19		40 860	1 417	1.14(0.98-1.32)	0.08		
≥once /wk	73 950	2 522	1.08(0.93-1.26)	0.33		99 497	3 547	1.15(1-1.33)	0.06		
Cheese					0.15					0.55	0.18

Never	2 265	76	1.00 (REF)			4 472	138	1.00 (REF)			
< once /wk	19 158	603	0.91(0.72-1.16)	0.44		30 983	1 105	1.14(0.95-1.36)	0.15		
≥once /wk	89 218	3 102	0.99(0.79-1.25)	0.94		108 264	3 804	1.13(0.95-1.34)	0.17		
Cooked vegetable					0.18					0.38	0.24
< 2 servings/d	18 763	651	1.00 (REF)			24 149	786	1.00 (REF)			
2.0-2.9 servings/d	39 170	1 370	0.99(0.90-1.08)	0.76		49 606	1 779	1.06(0.98-1.16)	0.15		
3.0-3.9 servings/d	31 445	1 035	0.92(0.84-1.02)	0.11		42 557	1 505	1.04(0.96-1.14)	0.34		
≥4 servings/d	23 302	780	0.95(0.86-1.06)	0.38		29 917	1 077	1.06(0.97-1.17)	0.20		
Raw vegetable					0.08					0.16	0.83
< 2 servings/d	42 219	1 487	1.00 (REF)			57 991	2 074	1.00 (REF)			
2.0-2.9 servings/d	29 300	988	0.95(0.88-1.03)	0.23		36 020	1 285	1.00(0.93-1.07)	0.90		
3.0-3.9 servings/d	18 121	628	0.98(0.89-1.08)	0.69		23 480	826	0.98(0.91-1.07)	0.68		
≥4 servings/d	23 015	735	0.91(0.83-1.00)	**0.04**		28 503	957	0.94(0.87-1.02)	0.14		
Fresh fruit					**<0.01**					**0.04**	0.06
< 2 servings/d	31 699	1 147	1.00 (REF)			45 409	1 573	1.00 (REF)			
2.0-2.9 servings/d	33 987	1 160	0.93(0.86-1.01)	0.09		42 206	1 529	1.02(0.95-1.09)	0.66		
3.0-3.9 servings/d	26 392	901	0.93(0.85-1.01)	0.10		33 387	1 210	1.01(0.93-1.09)	0.86		
≥4 servings/d	21 012	640	0.83(0.75-0.92)	**<0.01**		26 395	863	0.90(0.83-0.98)	**0.02**		
Dried fruit					0.82					0.07	0.56
< 2 servings/d	89 250	3 054	1.00 (REF)			119 255	4 216	1.00 (REF)			
2.0-2.9 servings/d	10 767	350	0.94(0.84-1.06)	0.31		11 979	429	0.99(0.90-1.09)	0.84		
3.0-3.9 servings/d	5 289	173	0.94(0.80-1.09)	0.42		6 070	192	0.87(0.75-1.01)	0.06		
≥4 servings/d	7 074	250	1.03(0.90-1.17)	0.68		8 769	294	0.93(0.82-1.04)	0.21		
Healthy dietary patterns					**<0.01**					0.75	0.07
poor dietary pattern	13 537	507	1.00 (REF)			19 186	665	1.00 (REF)			
medium dietary pattern	69 672	2 375	0.90(0.82-0.95)	**0.04**		90 544	3 184	1.00(0.92-1.09)	0.96		
ideal dietary pattern	28 819	937	0.86(0.77-0.99)	**<0.01**		34 568	1 220	0.99(0.90-1.09)	0.83		

Supplementary Table 7. The interaction between diet and genetic risk score on breast cancer risk.

	**GRS**				**ER+ GRS**				**ER- GRS**			
	Total No.	No. of cases	Haz Ratio (95%CI)	P value	Total No.	No. of cases	Haz Ratio (95%CI)	P value	Total No.	No. of cases	Haz Ratio (95%CI)	P value
Quartile 1												
Processed meat												
Never	7852	122	1.00 (REF)		7860	128	1.00 (REF)		7854	151	1.00 (REF)	
< once /wk	24023	**484**	**1.26(1.03-1.54)**	**0.02**	24013	474	**1.18(0.97-1.44)**	0.10	24003	570	**1.20(1.00-1.43)**	0.05
≥once /wk	31339	596	**1.20(0.98-1.46)**	0.07	31342	584	**1.13(0.93-1.37)**	0.21	31351	675	**1.10(0.92-1.32)**	0.29
Quartile 2												
Processed meat												
Never	7796	181	1.00 (REF)		7845	185	1.00 (REF)		7930	201	1.00 (REF)	
< once /wk	23872	652	**1.12(0.95-1.32)**	0.18	23890	642	**1.09(0.92-1.29)**	0.30	23905	722	**1.16(0.99-1.35)**	0.07
≥once /wk	31527	846	**1.12(0.95-1.32)**	0.16	31444	871	**1.14(0.97-1.34)**	0.10	31317	948	**1.17(1.00-1.36)**	0.05
Quartile 3												
Processed meat												
Never	7949	262	1.00 (REF)		7888	248	1.00 (REF)		8068	288	1.00 (REF)	
< once /wk	24202	909	**1.10(0.96-1.26)**	0.19	24172	913	**1.15(1.00-1.32)**	0.05	24135	946	**1.05(0.92-1.20)**	0.49
≥once /wk	30966	1184	**1.12(0.98-1.28)**	0.10	31071	1172	**1.15(1.00-1.32)**	0.05	30947	1154	**1.01(0.89-1.15)**	0.88
Quartile 4												
Processed meat												
Never	8206	419	1.00 (REF)		8210	423	1.00 (REF)		7951	344	1.00 (REF)	
< once /wk	23931	1330	**1.04(0.93-1.16)**	0.13	23953	1346	**1.05(0.94-1.17)**	0.42	23985	1137	**1.05(0.93-1.19)**	0.42
≥once /wk	30881	1762	**1.07(0.96-1.19)**	0.22	30856	1761	**1.07(0.96-1.19)**	0.23	31098	**1611**	**1.15(1.02-1.30)**	**0.02**
P for interaction				0.67				0.94				**0.03**
RERI			0.07(-0.33-0.46)	0.73			0.11(-0.26-0.49)	0.55			0.27(-0.03-0.57)	0.08
Quartile 1												
Beef												
Never	8408	169	1.00 (REF)		8384	170	1.00 (REF)		8353	182	1.00 (REF)	
< once /wk	29368	561	**0.90(0.76-1.07)**	0.24	29378	554	**0.88(0.74-1.05)**	0.16	29414	636	**0.94(0.80-1.11)**	0.50
≥once /wk	25304	467	**0.86(0.72-1.03)**	0.11	25324	**458**	**0.83(0.69-1.00)**	**0.04**	25284	577	**0.99(0.83-1.17)**	0.88
Quartile 2												
Beef												
Never	8423	189	1.00 (REF)		8513	201	1.00 (REF)		8492	245	1.00 (REF)	
< once /wk	29269	781	**1.11(0.95-1.31)**	0.20	29268	788	**1.07(0.91-1.25)**	0.41	29156	877	**0.98(0.85-1.13)**	0.76
≥once /wk	25381	705	**1.12(0.95-1.32)**	0.16	25278	702	**1.09(0.92-1.27)**	0.32	25424	744	**0.94(0.81-1.09)**	0.39

Quartile 3												
Beef												
Never	8614	291	1.00 (REF)		8613	273	1.00 (REF)		8759	298	1.00 (REF)	
< once /wk	28976	1089	**1.05(0.92-1.19)**	0.49	28949	1067	**1.10(0.96-1.25)**	0.19	29082	1103	**1.05(0.92-1.20)**	0.44
≥once /wk	25420	970	**1.06(0.92-1.21)**	0.42	25454	987	**1.15(1.00-1.31)**	0.05	25168	981	**1.08(0.95-1.24)**	0.23
Quartile 4												
Beef												
Never	8978	439	1.00 (REF)		8913	444	1.00 (REF)		8819	363	1.00 (REF)	
< once /wk	28939	1592	**1.06(0.95-1.18)**	0.31	28957	1614	**1.05(0.95-1.17)**	0.33	28900	1407	**1.12(1.00-1.26)**	0.05
≥once /wk	24993	**1473**	**1.13(1.02-1.26)**	**0.02**	25042	1468	**1.11(0.99-1.23)**	0.07	25222	**1313**	**1.19(1.05-1.34)**	**<0.01**
P for interaction				0.23				0.32				0.29
RERI			0.46(0.13-0.80)	**<0.01**			0.42(0.11-0.75)	**<0.01**			0.37(0.07-0.68)	**0.02**
Quartile 1												
Lamb												
Never	12913	235	1.00 (REF)		12880	234	1.00 (REF)		13019	283	1.00 (REF)	
< once /wk	35836	672	**0.98(0.84-1.14)**	0.81	35806	655	**0.96(0.82-1.11)**	0.58	35784	767	**0.94(0.82-1.08)**	0.37
≥once /wk	14233	290	**1.03(0.87-1.23)**	0.71	14306	293	**1.04(0.87-1.23)**	0.70	14151	346	**1.06(0.90-1.24)**	0.48
Quartile 2												
Lamb												
Never	13026	301	1.00 (REF)		13055	313	1.00 (REF)		12898	333	1.00 (REF)	
< once /wk	35728	979	**1.12(0.98-1.28)**	0.09	35724	987	**1.09(0.96-1.24)**	0.18	35779	**1113**	**1.15(1.02-1.31)**	**0.02**
≥once /wk	14231	391	**1.09(0.94-1.27)**	0.26	14182	388	**1.05(0.909-1.22)**	0.51	14261	413	**1.03(0.89-1.20)**	0.65
Quartile 3												
Lamb												
Never	12985	452	1.00 (REF)		12978	446	1.00 (REF)		13083	455	1.00 (REF)	
< once /wk	35480	1314	**1.03(0.92-1.14)**	0.63	35477	1303	**1.03(0.92-1.15)**	0.61	35451	1344	**1.03(0.93-1.15)**	0.59
≥once /wk	14383	577	**1.07(0.95-1.22)**	0.24	14419	574	**1.08(0.95-1.23)**	0.23	14359	575	**1.06(0.94-1.21)**	0.33
Quartile 4												
Lamb												
Never	12934	652	1.00 (REF)		12945	647	1.00 (REF)		12858	569	1.00 (REF)	
< once /wk	35195	2002	**1.08(0.99-1.18)**	0.09	35232	**2022**	**1.10(1.01-1.21)**	**0.03**	35225	1743	**1.09(0.99-1.19)**	0.09
≥once /wk	14645	849	**1.10(0.99-1.22)**	0.08	14585	**852**	**1.12(1.01-1.24)**	**0.04**	14721	**773**	**1.14(1.02-1.27)**	**0.02**
P for interaction				0.83				0.74				0.24
RERI			0.20(-0.16-0.56)	0.27			0.24(-0.11-0.59)	0.18			0.19(-0.07-0.46)	0.15
Quartile 1												
Pork												

Never	12481	231	1.00 (REF)		12473	228	1.00 (REF)		12502	262	1.00 (REF)	
< once /wk	36038	662	**0.95(0.81-1.10)**	0.48	35989	647	**0.94(0.81-1.10)**	0.46	36175	793	**0.99(0.86-1.14)**	0.93
≥once /wk	14446	301	**1.06(0.89-1.27)**	0.48	14510	304	**1.09(0.92-1.30)**	0.34	14274	340	**1.08(0.92-1.27)**	0.35
Quartile 2												
Pork												
Never	12524	311	1.00 (REF)		12575	322	1.00 (REF)		12551	341	1.00 (REF)	
< once /wk	36092	983	**1.03(0.91-1.17)**	0.63	36061	985	**1.01(0.89-1.14)**	0.92	35879	1084	**1.06(0.94-1.20)**	0.35
≥once /wk	14367	379	**0.99(0.85-1.15)**	0.91	14317	383	**0.97(0.83-1.13)**	0.70	14531	434	**1.03(0.89-1.19)**	0.72
Quartile 3												
Pork												
Never	12778	443	1.00 (REF)		12767	433	1.00 (REF)		13004	472	1.00 (REF)	
< once /wk	35516	1361	**1.05(0.95-1.18)**	0.34	35567	1365	**1.08(0.97-1.20)**	0.18	35224	1357	**1.00(0.90-1.11)**	0.98
≥once /wk	14609	541	**1.01(0.89-1.15)**	0.89	14582	526	**1.00(0.88-1.14)**	1.00	14699	551	**0.97(0.86-1.10)**	0.68
Quartile 4												
Pork												
Never	13361	672	1.00 (REF)		13329	674	1.00 (REF)		13087	582	1.00 (REF)	
< once /wk	34768	1990	**1.06(0.97-1.16)**	0.17	34797	1999	**1.07(0.98-1.16)**	0.16	35136	1762	**1.07(0.97-1.18)**	0.16
≥once /wk	14656	838	**1.06(0.96-1.18)**	0.25	14669	846	**1.07(0.97-1.19)**	0.18	14574	734	**1.06(0.95-1.19)**	0.29
P for interaction				0.47				0.45				0.67
RERI			0.14(-0.19-0.48)	0.40			0.14(-0.22-0.50)	0.44			0.07(-0.22-0.37)	0.63
Quartile 1												
Poultry												
Never	3891	65	1.00 (REF)		3890	70	1.00 (REF)		3857	75	1.00 (REF)	
< once /wk	6577	131	**1.10(0.81-1.48)**	0.55	6538	129	**1.01(0.75-1.35)**	0.97	6566	159	**1.16(0.88-1.53)**	0.29
≥once /wk	52740	1006	**1.08(0.83-1.39)**	0.57	52779	987	**0.98(0.76-1.25)**	0.84	52789	1165	**1.07(0.85-1.36)**	0.55
Quartile 2												
Poultry												
Never	3916	75	1.00 (REF)		3925	80	1.00 (REF)		3883	96	1.00 (REF)	
< once /wk	6423	**183**	**1.38(1.06-1.81)**	0.02	6502	177	**1.23(0.94-1.60)**	0.13	6474	177	**1.02(0.80-1.31)**	0.86
≥once /wk	52876	**1421**	**1.29(1.02-1.63)**	0.03	52785	1442	**1.25(0.99-1.56)**	0.06	52819	1597	**1.15(0.94-1.42)**	0.18
Quartile 3												
Poultry												
Never	3828	127	1.00 (REF)		3835	120	1.00 (REF)		4045	136	1.00 (REF)	
< once /wk	6549	252	**1.08(0.87-1.34)**	0.48	6501	255	**1.17(0.94-1.46)**	0.16	6452	261	**1.14(0.92-1.40)**	0.23
≥once /wk	52762	1976	**1.08(0.90-1.29)**	0.43	52812	1957	**1.12(0.93-1.35)**	0.25	52684	1988	**1.07(0.90-1.28)**	0.45
Quartile 4												

Poultry												
Never	4021	205	1.00 (REF)		4006	202	1.00 (REF)		3871	165	1.00 (REF)	
< once /wk	6405	351	**1.00(0.84-1.19)**	0.97	6413	356	**1.03(0.87-1.23)**	0.74	6462	320	**1.08(0.89-1.30)**	0.43
≥once /wk	52687	2963	**1.05(0.91-1.22)**	0.47	52689	2980	**1.08(0.93-1.24)**	0.32	52773	2616	**1.10(0.94-1.29)**	0.23
P for interaction				0.50				0.63				0.75
RERI			0.09(-0.50-0.67)	0.78			0.21(-0.27-0.71)	0.39			0.15(-0.23-0.54)	0.44
Quartile 1												
Oily fish												
Never	6710	123	1.00 (REF)		6689	122	1.00 (REF)		6789	143	1.00 (REF)	
< once /wk	20094	373	**0.97(0.79-1.19)**	0.74	20198	369	**0.95(0.77-1.16)**	0.60	20098	475	**1.06(0.88-1.28)**	0.54
≥once /wk	36269	706	**0.98(0.81-1.20)**	0.86	36187	695	**0.96(0.79-1.16)**	0.66	36157	778	**0.94(0.78-1.12)**	0.48
Quartile 2												
Oily fish												
Never	6778	143	1.00 (REF)		6789	149	1.00 (REF)		6637	152	1.00 (REF)	
< once /wk	20184	**549**	**1.21(1.00-1.46)**	**0.04**	20078	550	**1.18(0.98-1.42)**	0.07	20288	**589**	**1.23(1.03-1.48)**	**0.02**
≥once /wk	36040	981	**1.16(0.97-1.39)**	0.10	36113	994	**1.15(0.96-1.37)**	0.13	36056	**1124**	**1.29(1.09-1.54)**	**<0.01**
Quartile 3												
Oily fish												
Never	6774	220	1.00 (REF)		6823	225	1.00 (REF)		6952	234	1.00 (REF)	
< once /wk	20175	764	**1.14(0.98-1.32)**	0.10	20181	772	**1.13(0.98-1.32)**	0.10	20020	752	**1.06(0.91-1.23)**	0.46
≥once /wk	35975	1364	**1.11(0.96-1.29)**	0.15	35953	1328	**1.07(0.93-1.23)**	0.37	35988	1396	**1.06(0.92-1.22)**	0.42
Quartile 4												
Oily fish												
Never	6845	348	1.00 (REF)		6806	338	1.00 (REF)		6729	305	1.00 (REF)	
< once /wk	20082	1108	**1.05(0.93-1.18)**	0.48	20078	1103	**1.07(0.94-1.20)**	0.31	20129	978	**1.04(0.92-1.19)**	0.53
≥once /wk	35935	**2049**	**1.05(0.94-1.18)**	0.38	35966	2083	**1.09(0.97-1.23)**	0.14	36018	1802	**1.04(0.92-1.18)**	0.50
P for interaction				0.86				0.61				0.26
RERI			0.16(-0.15-0.48)	0.31			0.28(-0.04-0.61)	0.08			0.12(-0.16-0.40)	0.40
Quartile 1												
Non-oily fish												
Never	2994	58	1.00 (REF)		2987	61	1.00 (REF)		3060	56	1.00 (REF)	
< once /wk	17689	318	**0.89(0.67-1.18)**	0.41	17671	314	**0.83(0.63-1.09)**	0.18	17715	395	**1.16(0.87-1.53)**	0.31
≥once /wk	42400	827	**0.95(0.73-1.24)**	0.71	42425	812	**0.87(0.67-1.14)**	0.31	42289	947	**1.15(0.88-1.51)**	0.30
Quartile 2												
Non-oily fish												
Never	3074	62	1.00 (REF)		3090	62	1.00 (REF)		2960	76	1.00 (REF)	

< once /wk	17818	480	**1.24(0.95-1.62)**	0.11	17482	491	**1.29(0.99-1.68)**	0.06	18067	533	**1.09(0.86-1.39)**	0.48
≥once /wk	42163	1133	**1.21(0.93-1.57)**	0.15	42105	1141	**1.25(0.96-1.62)**	0.09	42013	1257	**1.09(0.86-1.37)**	0.49
Quartile 3												
Non-oily fish												
Never	3097	89	1.00 (REF)		3144	92	1.00 (REF)		3210	99	1.00 (REF)	
< once /wk	18107	**709**	**1.31(1.05-1.63)**	**0.02**	18082	**686**	**1.25(1.01-1.56)**	**0.04**	17867	655	**1.13(0.92-1.40)**	0.25
≥once /wk	41786	1554	**1.23(0.99-1.53)**	0.06	41803	1552	**1.21(0.98-1.50)**	0.08	41942	1629	**1.18(0.97-1.45)**	0.10
Quartile 4												
Non-oily fish												
Never	3216	156	1.00 (REF)		3160	150	1.00 (REF)		3151	134	1.00 (REF)	
< once /wk	17932	975	**1.07(0.90-1.26)**	0.45	17951	991	**1.10(0.93-1.31)**	0.27	17897	899	**1.13(0.94-1.35)**	0.20
≥once /wk	41712	2366	**1.08(0.92-1.27)**	0.34	41728	2375	**1.11(0.94-1.31)**	0.23	41817	2047	**1.07(0.90-1.28)**	0.42
P for interaction				0.32				0.32				0.73
RERI			0.28(-0.23-0.80)	0.28			0.40(-0.06-0.86)	0.09			0.02(-0.52-0.55)	0.94
Quartile 1												
Cheese												
Never	1552	28	1.00 (REF)		1551	27	1.00 (REF)		1534	34	1.00 (REF)	
< once /wk	11736	213	**1.01(0.68-1.51)**	0.94	11762	212	**1.03(0.69-1.55)**	0.87	11819	253	**0.94(0.66-1.35)**	0.75
≥once /wk	48464	940	**1.10(0.75-1.60)**	0.64	48415	923	**1.10(0.75-1.62)**	0.62	48353	1085	**1.00(0.71-1.40)**	0.98
Quartile 2												
Cheese												
Never	1541	40	1.00 (REF)		1553	44	1.00 (REF)		1588	35	1.00 (REF)	
< once /wk	11972	317	**0.95(0.69-1.33)**	0.78	11992	333	**0.93(0.68-1.28)**	0.68	11985	332	**1.22(0.86-1.72)**	0.27
≥once /wk	48127	1286	**0.97(0.71-1.33)**	0.85	48120	1285	**0.91(0.67-1.23)**	0.55	48023	1464	**1.34(0.96-1.87)**	0.09
Quartile 3												
Cheese												
Never	1652	53	1.00 (REF)		1652	49	1.00 (REF)		1593	66	1.00 (REF)	
< once /wk	12075	430	**1.08(0.81-1.44)**	0.59	12103	399	**1.09(0.81-1.46)**	0.59	12086	468	**0.91(0.70-1.18)**	0.48
≥once /wk	47848	1812	**1.15(0.88-1.52)**	0.31	47813	1827	**1.27(0.95-1.69)**	0.10	47954	1796	**0.87(0.68-1.12)**	0.28
Quartile 4												
Cheese												
Never	1708	88	1.00 (REF)		1697	89	1.00 (REF)		1738	74	1.00 (REF)	
< once /wk	12673	675	**1.00(0.80-1.25)**	0.98	12599	691	**1.01(0.81-1.26)**	0.93	12566	582	**1.05(0.82-1.34)**	0.70
≥once /wk	47002	2666	**1.04(0.84-1.29)**	0.71	47093	2669	**1.02(0.82-1.26)**	0.89	47111	2359	**1.13(0.89-1.42)**	0.32
P for interaction				0.98				0.56				0.46
RERI			0.07(-0.75-0.89)	0.86			0.01(-0.85-0.87)	0.98			0.23(-0.34-0.81)	0.43

Quartile 1												
Cooked vegetable												
< 2 servings/d	10113	181	1.00 (REF)		10096	175	1.00 (REF)		10291	229	1.00 (REF)	
2.0-2.9 servings/d	21945	425	**1.06(0.89-1.27)**	0.50	21994	419	**1.07(0.90-1.28)**	0.44	21658	501	**1.01(0.86-1.18)**	0.90
3.0-3.9 servings/d	18234	357	**1.07(0.89-1.28)**	0.50	18127	356	**1.10(0.92-1.32)**	0.30	18175	378	**0.91(0.77-1.08)**	0.27
≥4 servings/d	12504	235	**1.03(0.85-1.25)**	0.77	12568	231	**1.04(0.85-1.27)**	0.71	12629	282	**0.98(0.82-1.18)**	0.86
Quartile 2												
Cooked vegetable												
< 2 servings/d	10386	260	1.00 (REF)		10444	282	1.00 (REF)		10204	285	1.00 (REF)	
2.0-2.9 servings/d	21547	584	**1.02(0.88-1.19)**	0.75	21531	576	**0.94(0.82-1.09)**	0.42	21622	636	**1.03(0.89-1.18)**	0.73
3.0-3.9 servings/d	18028	475	**1.00(0.86-1.17)**	0.98	18007	478	**0.94(0.81-1.09)**	0.41	18011	544	**1.04(0.90-1.20)**	0.61
≥4 servings/d	12784	345	**1.03(0.87-1.21)**	0.75	12751	350	**0.97(0.83-1.14)**	0.70	12897	397	**1.06(0.91-1.24)**	0.45
Quartile 3												
Cooked vegetable												
< 2 servings/d	10365	397	1.00 (REF)		10367	380	1.00 (REF)		10419	365	1.00 (REF)	
2.0-2.9 servings/d	21645	817	**0.97(0.86-1.09)**	0.60	21584	808	**1.00(0.89-1.14)**	0.95	21796	878	**1.09(0.97-1.24)**	0.15
3.0-3.9 servings/d	17675	660	**0.95(0.84-1.08)**	0.42	17779	666	**1.00(0.88-1.14)**	0.99	17698	656	**1.00(0.88-1.14)**	0.99
≥4 servings/d	12978	469	**0.92(0.80-1.06)**	0.25	12946	466	**0.97(0.84-1.11)**	0.62	12787	468	**1.00(0.87-1.15)**	0.98
Quartile 4												
Cooked vegetable												
< 2 servings/d	10615	550	1.00 (REF)		10572	551	1.00 (REF)		10565	509	1.00 (REF)	
2.0-2.9 servings/d	20936	1227	**1.08(0.97-1.19)**	0.15	20964	1250	**1.09(0.99-1.21)**	0.09	20997	1038	**1.00(0.89-1.11)**	0.93
3.0-3.9 servings/d	17799	963	**0.98(0.88-1.09)**	0.71	17823	955	**0.96(0.87-1.07)**	0.50	17852	877	**0.98(0.87-1.09)**	0.66
≥4 servings/d	13187	750	**1.06(0.95-1.19)**	0.31	13188	752	**1.05(0.94-1.18)**	0.35	13140	652	**1.00(0.89-1.13)**	1.00
P for interaction				0.88				0.72				0.80
RERI			0.20(-0.17-0.57)	0.29			0.18(-0.21-0.57)	0.37			0.03(-0.26-0.33)	0.83
Quartile 1												
Raw vegetable												
< 2 servings/d	24348	461	1.00 (REF)		24378	461	1.00 (REF)		24379	563	1.00 (REF)	
2.0-2.9 servings/d	16089	308	**1.02(0.88-1.18)**	0.81	15998	298	**0.99(0.86-1.15)**	0.91	15918	331	**0.90(0.79-1.03)**	0.14
3.0-3.9 servings/d	10064	209	**1.10(0.93-1.30)**	0.26	10071	195	**1.03(0.87-1.21)**	0.77	9974	236	**1.03(0.88-1.20)**	0.71
≥4 servings/d	12240	219	**0.96(0.81-1.13)**	0.61	12277	228	**1.00(0.85-1.17)**	0.97	12405	258	**0.90(0.78-1.05)**	0.19
Quartile 2												
Raw vegetable												
< 2 servings/d	24324	682	1.00 (REF)		24254	686	1.00 (REF)		24117	713	1.00 (REF)	
2.0-2.9 servings/d	15756	398	**0.89(0.79-1.01)**	0.07	15817	**396**	**0.88(0.78-0.99)**	**0.04**	15918	478	**1.01(0.90-1.14)**	0.83
3.0-3.9 servings/d	10062	265	**0.93(0.81-1.08)**	0.34	10078	290	**1.01(0.88-1.16)**	0.87	10088	295	**0.98(0.86-1.13)**	0.78

≥4 servings/d	12507	314	**0.89(0.77-1.02)**	0.08	12506	**308**	**0.86(0.75-0.99)**	**0.03**	12537	370	**0.99(0.87-1.13)**	0.89
Quartile 3												
Raw vegetable												
< 2 servings/d	24203	927	1.00 (REF)		24309	930	1.00 (REF)		24267	930	1.00 (REF)	
2.0-2.9 servings/d	15773	590	**0.98(0.88-1.09)**	0.71	15737	589	**0.98(0.89-1.09)**	0.77	15779	606	**1.00(0.90-1.10)**	0.93
3.0-3.9 servings/d	10111	373	**0.97(0.86-1.09)**	0.60	10188	363	**0.94(0.83-1.06)**	0.31	10082	403	**1.04(0.93-1.17)**	0.47
≥4 servings/d	12529	453	**0.96(0.86-1.07)**	0.86	12384	435	**0.94(0.84-1.05)**	0.28	12516	435	**0.92(0.82-1.03)**	0.17
Quartile 4												
Raw vegetable												
< 2 servings/d	24173	1381	1.00 (REF)		24107	1374	1.00 (REF)		24285	1245	1.00 (REF)	
2.0-2.9 servings/d	15671	896	**0.99(0.91-1.08)**	0.87	15737	90	**1.00(0.92-1.09)**	0.91	15674	777	**0.97(0.88-1.06)**	0.44
3.0-3.9 servings/d	10058	557	**0.97(0.88-1.07)**	0.54	9958	556	**0.98(0.89-1.08)**	0.65	10151	470	**0.90(0.81-1.00)**	0.05
≥4 servings/d	12575	661	**0.93(0.85-1.02)**	0.15	12684	676	**0.94(0.86-1.04)**	0.23	12393	584	**0.93(0.84-1.03)**	0.17
P for interaction				0.83				0.62				0.63
RERI			-0.16(-0.49-0.28)	0.36			-0.15(-0.50-0.20)	0.40			-0.06(-0.34-0.21)	0.66
Quartile 1												
Fresh fruit												
< 2 servings/d	18666	368	1.00 (REF)		18768	360	1.00 (REF)		18668	418	1.00 (REF)	
2.0-2.9 servings/d	18563	359	**0.97(0.84-1.12)**	0.66	18573	355	**0.97(0.84-1.13)**	0.73	18501	421	**1.00(0.87-1.14)**	0.94
3.0-3.9 servings/d	14399	280	**0.96(0.82-1.13)**	0.64	14341	280	**0.98(0.84-1.15)**	0.80	14442	324	**0.96(0.83-1.12)**	0.63
≥4 servings/d	11499	195	**0.84(0.70-1.00)**	0.06	11441	192	**0.84(0.70-1.00)**	0.05	11493	232	**0.87(0.74-1.03)**	0.10
Quartile 2												
Fresh fruit												
< 2 servings/d	18607	503	1.00 (REF)		18487	531	1.00 (REF)		18503	540	1.00 (REF)	
2.0-2.9 servings/d	18589	500	**0.95(0.84-1.08)**	0.42	18571	493	**0.89(0.78-1.00)**	0.06	18474	543	**0.99(0.88-1.12)**	0.85
3.0-3.9 servings/d	14625	396	**0.94(0.82-1.07)**	0.35	14722	391	**0.87(0.76-1.00)**	0.04	14599	452	**1.05(0.93-1.19)**	0.45
≥4 servings/d	11288	**275**	**0.83(0.72-0.97)**	**0.02**	11325	**277**	**0.79(0.68-0.92)**	**<0.01**	11501	334	**0.97(0.85-1.12)**	0.69
Quartile 3												
Fresh fruit												
< 2 servings/d	18680	713	1.00 (REF)		18709	686	1.00 (REF)		18682	749	1.00 (REF)	
2.0-2.9 servings/d	18394	687	**0.97(0.87-1.07)**	0.52	18395	693	**1.01(0.91-1.13)**	0.79	18493	**673**	**0.86(0.78-0.96)**	**0.01**
3.0-3.9 servings/d	14491	569	**1.01(0.90-1.13)**	0.86	14457	572	**1.06(0.95-1.19)**	0.31	14495	577	**0.93(0.83-1.04)**	0.19
≥4 servings/d	11476	**383**	**0.86(0.75-0.97)**	**0.02**	11497	380	**0.88(0.78-1.00)**	0.06	11409	**380**	**0.78(0.68-0.88)**	**<0.01**
Quartile 4												
Fresh fruit												
< 2 servings/d	18643	1068	1.00 (REF)		18632	1075	1.00 (REF)		18743	945	1.00 (REF)	
2.0-2.9 servings/d	18295	1048	**0.97(0.89-1.06)**	0.49	18302	1053	**0.97(0.89-1.06)**	0.47	18373	957	**1.02(0.93-1.12)**	0.66

3.0-3.9 servings/d	14438	794	**0.92(0.84-1.01)**	0.10	14433	796	**0.92(0.84-1.01)**	0.09	14417	686	**0.92(0.84-1.02)**	0.12
≥4 servings/d	11602	**596**	**0.87(0.78-0.96)**	**<0.01**	11602	**600**	**0.87(0.78-0.96)**	**0.01**	11462	**503**	**0.85(0.76-0.95)**	**<0.01**
P for interaction				1.00				0.81				0.34
RERI			-0.23(-0.55-0.09)	0.15			-0.26(-0.61-0.08)	0.14			-0.24(-0.52-0.03)	0.08
Quartile 1												
Dried fruit												
< 2 servings/d	50420	966	1.00 (REF)		50410	955	1.00 (REF)		50485	1116	1.00 (REF)	
2.0-2.9 servings/d	5610	93	**0.85(0.69-1.05)**	0.14	5628	93	**0.85(0.69-1.05)**	0.14	5501	112	**0.91(0.75-1.10)**	0.33
3.0-3.9 servings/d	2812	52	**0.93(0.71-1.24)**	0.63	2797	56	**1.01(0.77-1.33)**	0.92	2784	61	**0.96(0.74-1.24)**	0.75
≥4 servings/d	3826	77	**1.03(0.81-1.30)**	0.83	3831	70	**0.93(0.73-1.18)**	0.54	3868	89	**1.04(0.84-1.29)**	0.73
Quartile 2												
Dried fruit												
< 2 servings/d	50702	1348	1.00 (REF)		50623	1357	1.00 (REF)		50525	1524	1.00 (REF)	
2.0-2.9 servings/d	5444	141	**0.95(0.79-1.13)**	0.53	5467	142	**0.95(0.80-1.13)**	0.55	5543	145	**0.85(0.71-1.00)**	0.06
3.0-3.9 servings/d	2681	79	**1.07(0.85-1.34)**	0.56	2692	74	**1.00(0.79-1.26)**	0.99	2752	81	**0.96(0.76-1.20)**	0.70
≥4 servings/d	3815	99	**0.94(0.77-1.16)**	0.56	3852	114	**1.08(0.89-1.31)**	0.42	3766	109	**0.94(0.77-1.14)**	0.50
Quartile 3												
Dried fruit												
< 2 servings/d	50524	1930	1.00 (REF)		50613	1913	1.00 (REF)		50520	1937	1.00 (REF)	
2.0-2.9 servings/d	5500	188	**0.89(0.76-1.03)**	0.12	5435	184	**0.89(0.76-1.03)**	0.12	5526	201	**0.92(0.80-1.07)**	0.28
3.0-3.9 servings/d	2741	82	**0.78(0.63-0.98)**	0.03	2763	87	**0.83(0.67-1.03)**	0.09	2728	**87**	**0.80(0.64-0.99)**	**0.04**
≥4 servings/d	3801	137	**0.94(0.79-1.12)**	0.50	3759	130	**0.91(0.76-1.09)**	0.30	3844	139	**0.92(0.77-1.09)**	0.34
Quartile 4												
Dried fruit												
< 2 servings/d	50248	2797	1.00 (REF)		50248	2816	1.00 (REF)		50364	2464	1.00 (REF)	
2.0-2.9 servings/d	5473	323	**1.05(0.94-1.18)**	0.39	5497	326	**1.05(0.93-1.17)**	0.44	5457	287	**1.07(0.95-1.21)**	0.27
3.0-3.9 servings/d	2778	144	**0.91(0.77-1.07)**	0.25	2760	140	**0.88(0.74-1.04)**	0.14	2748	128	**0.94(0.78-1.12)**	0.49
≥4 servings/d	3921	217	**0.99(0.86-1.14)**	0.87	3921	216	**0.98(0.85-1.12)**	0.74	3885	193	**1.01(0.87-1.17)**	0.88
P for interaction				0.33				0.53				0.29
RERI			-0.05(-0.55-0.45)	0.83			-0.01(-0.50-0.48)	0.97			-0.01(-0.41-0.38)	0.95

Multivariable Cox regression model adjusted for age at recruitment,smoking, ethnicity, physical activity level, townsend deprivation index, alcohol intake frequency, employment status, educational qualifications, BMI, 22 UKB centers, number of births, age at menarche, menopausal status, age at first birth, ever use of oral contraceptive pill use, ever use of hormone replacement therapy, family history of breast cancer, stratified by quartile of PRS. Two-way interaction between food-group intakes and polygenic risk score was evaluated by including the interaction term to the Cox models.
