# Supplementary figures and images for: The interaction of diet, alcohol, genetic predisposition, and the risk of breast cancer: a cohort study from the UK Biobank
Source: Eur J Nutr. 2023 Nov 1;63(2):343–56. doi: 10.1007/s00394-023-03269-8 (PMC10899287; doi:10.1007/s00394-023-03269-8)

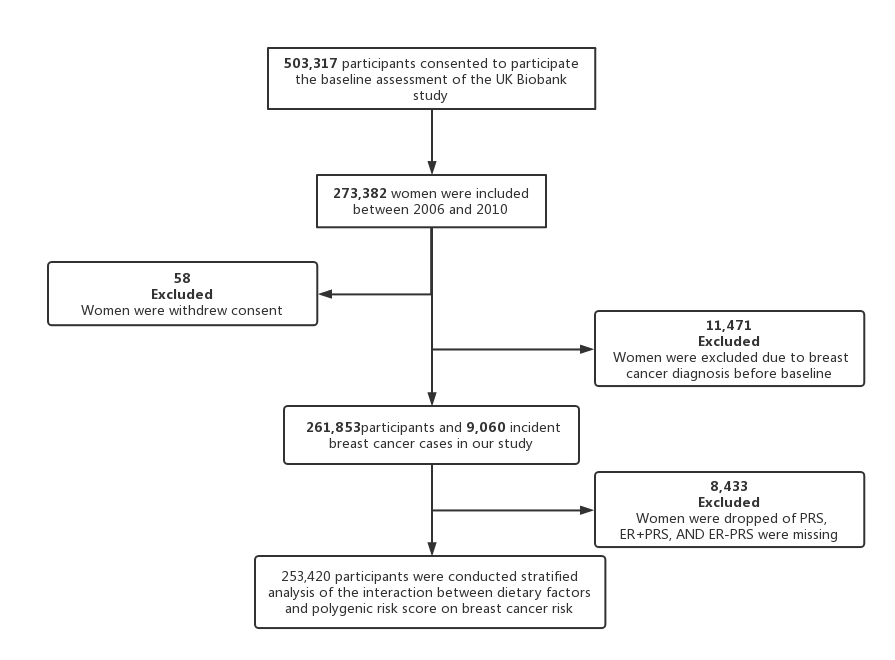

Supplement: Supplementary file 2 — Supplementary file2 (DOCX 112 KB) [file 394_2023_3269_MOESM2_ESM.docx]
